# Structure Elucidation of Two Intriguing Neo-Debromoaplysiatoxin Derivatives from Marine Cyanobacterium *Lyngbya* sp. Showing Strong Inhibition of Kv1.5 Potassium Channel and Differential Cytotoxicity

**DOI:** 10.3390/molecules28062786

**Published:** 2023-03-20

**Authors:** Zijun Chen, Na Chen, Peng Fu, Weiping Wang, Shilin Bian, Huihui Zhang, Sicheng Shen, Bingnan Han

**Affiliations:** 1 Department of Development Technology of Marine Resources, College of Life Sciences and Medicine, Zhejiang Sci-Tech University, Hangzhou 310018, China; 2 Key Laboratory of Marine Drugs, Ministry of Education of China, School of Medicine and Pharmacy, Ocean University of China, Qingdao 266003, China; 3 Institute of Materia Medica, Chinese Academy of Medical Sciences and Peking Union Medical College, Beijing 100730, China

**Keywords:** cyanobacteria, *Lyngbya* sp., aplysiatoxins, peroxide bridge, DP4+, Kv1.5 inhibition, cytotoxicity

## Abstract

Two aplysiatoxin derivatives, neo-debromoaplysiatoxin I (**1**) and neo-debromoaplysiatoxin J (**2**), were isolated from marine cyanobacterium *Lyngbya* sp. collected from the South China Sea. Their structures including absolute configurations were assigned by spectroscopic analysis, in combination with GIAO NMR shift calculation and DP4+ analysis. Structures of neo-debromoaplysiatoxin I and neo-debromoaplysiatoxin J contained a decahydro-5*H*-pyrano [2,3,4-de] chromen-5-one 6/6/6 ring skeleton and an intriguing peroxide bridge group, respectively, which are unprecedented structure scaffold and motif in aplysiatoxins. Two compounds displayed comparable inhibitory activities against Kv1.5 K^+^ channel with IC_50_ values of 2.59 ± 0.37 μM (**1**) and 1.64 ± 0.15 μM (**2**); however, they presented differential cytotoxic effects. It is worth noting that neo-debromoaplysiatoxin J, containing a peroxide bridge, showed remarkable cytotoxicity against four cancer cell lines including SW480, SGC7901, LoVo and PC-9 compared to the human normal cell line.

## 1. Introduction

Cyanobacteria (blue-green algae), known as one of the oldest bacteria in evolutionary history, are pioneers to conquer the ancient environments [1,2]. They are widely distributed in oceans, tropical reefs, freshwater ponds, rivers, polar ice and terrestrial substrates, among which the ocean is preferred due to its alkaline environment [3]. They have the ability to produce significant quantities of toxins—cyanotoxins, such as saxitoxins (consisting of a tetrahydropurine group and two guanidinium moieties), ciguatoxins (polyether) [4], microcystins (cyclic peptide) [5], lipopolysaccharide endotoxins [6] and so on. Aplysiatoxins (ATXs) are polyketides existing in cyanobacteria with anti-proliferative, antiviral, pro-inflammatory and Kv1.5 K^+^ channel inhibition properties [7,8,9,10]. Up to now, there have been about 55 aplysiatoxins isolated from cyanobacteria [11,12,13,14]. Based on structural skeletons, we classified them into four categories: 6/12/6 fused ring system featuring a macrolactone ring; spirobicyclic system; linear structure; and some neo-ATXs displaying uncommon carbon skeletons. Several ATXs were found to be activators of protein kinase C (PKC) [15,16], a vital therapeutic target for cancers [17], such as debromoaplysiatoxin (DAT) and 3-deoxy-DAT, probably owing to the dihydroxyvaleryl moiety in their structures, namely a recognition domain interacting with the PKC*δ* C1B domain in the form of hydrogen bonds [18]. In our previous studies, the inhibitory activity on the shaker-related subfamily of voltage-gated channels (Kv1.1–Kv1.5) was investigated, and potential drug targets for the treatment of diverse disease processes, ranging from cancer to autoimmune diseases to metabolic, neurological and cardiovascular disorders [19], of ATXs were tested, and it turned out several ATXs had selective and strong blocking inhibitory effects on the Kv1.5 K^+^ channel [10,11,12,20,21,22]. Among them, neo-debromoaplysiatoxin B (Neo-B) and oscillatoxin E displayed the strongest Kv1.5 inhibition activities with IC_50_ values of 0.3 μM and 0.79 μM respectively.

**Figure 1:**
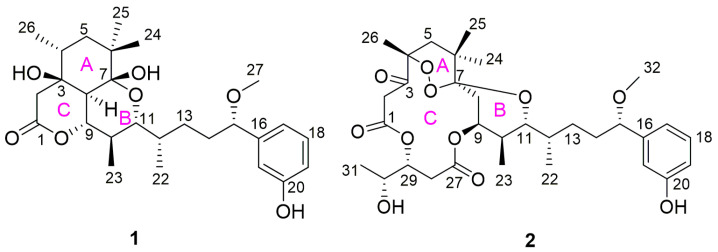
Structures of neo-debromoaplysiatoxin I (**1**) and neo-debromoaplysiatoxin J (**2**).

Recently, in our ongoing search for new ATXs from cyanobacteria, two undescribed ATX analogues, neo-debromoaplysiatoxin I (**1**) and neo-debromoaplysiatoxin J (**2**), were isolated (Figure 1). Compound **1** possessed decahydro-*5H*-pyrano [2,3,4-de] chromen-5-one structural skeleton, and compound **2** had a 6/14/6 fused-ring system. Although the ABC tricyclic ring system with a macrolide is quite common in ATXs, this is the first example of the introduction of a peroxide bridge in **2**. Their inhibitory activities against the Kv1.5 K^+^ channel and cytotoxic effects against human normal and cancer cell lines were evaluated. Herein, we report the isolation, structure elucidation and bioactivities of these two compounds.

## 2. Results and Discussion

### 2.1. Structure Elucidation of Compounds

Neo-debromoaplysiatoxin I (**1**) was obtained as a white solid, with a molecular formula of C_27_H_40_O_7_ based on its HRESIMS data (*m*/*z* 499.2668 [M + Na]^+^, calcd. for C_27_H_40_O_7_Na, 499.2672), requiring eight degrees of unsaturation. Compound **1** showed IR absorption bands for hydroxy group (3418 (br), 3260 (br) cm^−1^), ester linkage (1712 cm^−1^) and aromatic system (1663, 1646, 1455 cm^−1^). The ^13^C and DEPT NMR spectra revealed 27 carbon signals, attributed to 6 quaternary carbons, 11 methines, 4 methylenes and 6 methyls including 1 methoxy (Table 1). Detailed ^1^H, ^13^C and HSQC NMR spectra analysis suggested the presence of a 1,3-disubstituted benzene ring, implying it might belong to ATXs.

**Figure 2:**
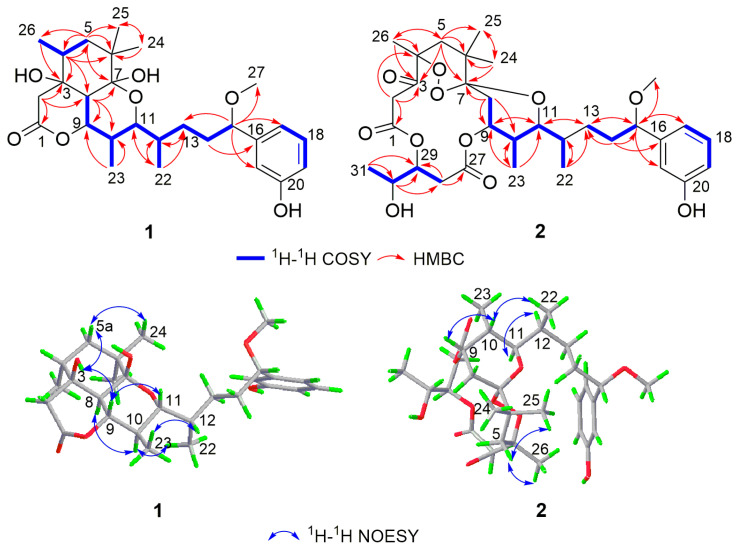
Key 2D correlations of **1** and **2**.

With an ester bond and a benzene ring taking five degrees of unsaturation, **1** contained a tricyclic core. The ring A was established by the HMBC correlations from H_3_-26 to C-3, C-4, C-5 and C-8; from H_3_-24, H_3_-25 to C-5, C-6 and C-7; from H_2_-5 to C-4 and C-6; and from H-8 to C-3, C-7 and C-9. The ^1^H-^1^H COSY correlations of H-4/H_2_-5/H_3_-26 supported the structure segment. 3-OH (*δ*_H_ 4.35, d, *J* = 2.0 Hz) exhibited HMBC correlation to C-2, COSY correlation with H-2a and the chemical shifts of C-1 (*δ*_C_ 170.5), C-2 (*δ*_C_ 40.9), C-3 (*δ*_C_ 72.6) and C-9 (*δ*_C_ 77.6), together with HMBC spectrum signals from H_2_-2 to C-1, C-3 and C-8, indicated that the ring C was composed of C-1, C-2, C-3, C-8 and C-9 through an ester bond to form a six-membered lactone ring with a hydroxy on C-3. Furthermore, IR spectrum showed absorption band at 1712 cm^−1^, which supported a six-membered lactone ring. As shown in Figure 2, the COSY signals of H-8/H-9/H-10/H_3_-23/H-11/H-12/H_3_-22/H_2_-13/H_2_-14/H-15 and HMBC correlations from H_3_-23 to C-9, C-10 and C-11; from H_3_-22 to C-11, C-12 and C-13; and from H-15 to C-14, C-13, C-17, C-21 and C-27 illustrated the structure segments from C-8 to C-21. The ring B was closed by C7-O-C11, and a hydroxy was attached to C-7 based on the ^13^C NMR chemical shifts of oxygenated quaternary carbon C-7 (*δ*_C_ 100.1), oxygenated tertiary carbon C-11 (*δ*_C_ 75.5) and unsaturation of **1**, as well as structural features of ATXs. Thereby, the planar structure of compound **1** was completed.

Its relative stereochemistry was established by the NOESY experiments and vicinal coupling constants. The key NOE correlations of 3-OH/H-5a, 3-OH/H-9, H-5a/H_3_-24 and H-5b/H-25/H-26 implicated 3-OH/H-4/H-5a/H_3_-24/H-9 shared *syn* relationships, while H-5b/H_3_-26/H_3_-25 presented same orientation. The small couplings of H-4 to H-5a and H-5b (*J*_5a,4_ = 5.8 Hz, *J*_5b,4_ = 2.1 Hz) determined H-4 as the equatorial bond of ring A, which exhibited the opposite configuration with H-4 of Neo-B ((*J*_5a,4_ = 6.8 Hz) [23]. The NOE cross-peak of H-8/H-10/H_3_-22, H-9/H-11/H_3_-23 defined an anti-relationship between H-8 and H-9, H-9 and H-10 and H-10 and H-11. The large coupling constants of H-9 (*J*_8,9_ = *J*_9,10_ = 10.7 Hz) and H-11 (*J*_10,11_ = 10.4 Hz) showed that H-8, 9, 10 and 11 were axially oriented. A *gauche* relationship of H-11 and H-12 was deduced from their NOESY correlation and small coupling (*J*_11,12_ = 1.9Hz). In addition, the NOE correlations of H-12/H_3_-23 and H-10/H_3_-22 shown in Figure 2 also clarified the rationality of this conformation. Agreements of ^1^H and ^13^C chemical shifts of C-15 between **1** and other ATXs, such as oscillatoxin D, oscillatoxin F [8] and 3-methoxydebromoaplysiatoxin [7], together with structural features and common biosynthetic origin of ATXs, suggested that the absolute stereochemistry of C-15 was identified as *S*. In Chembio3D, the three rings exhibited stable chair conformation when C-7 was *R*, while they showed boat conformation when C-7 was *S*.

**Figure 3:**
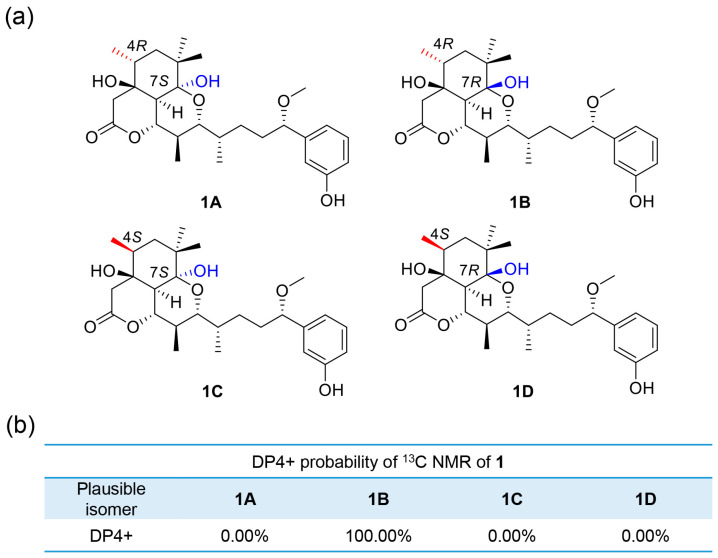
(**a**) Possible isomers for **1**. (**b**) DP4+ probability of ^13^C values of **1**.

**Figure 4:**
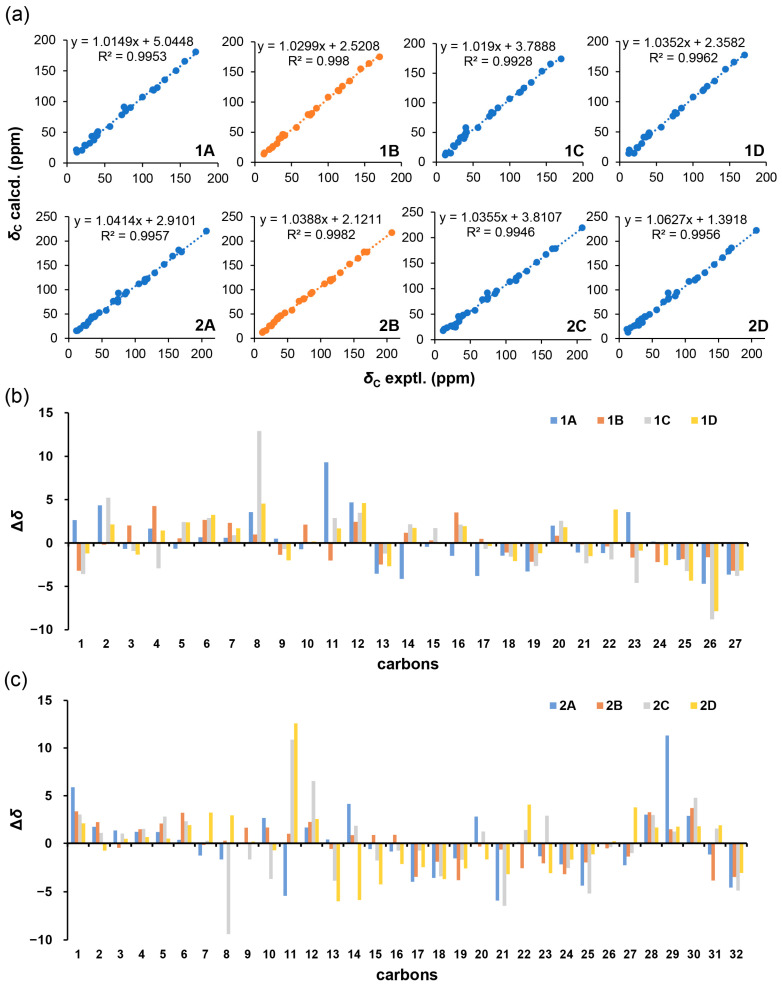
^13^C NMR calculation results of plausible epimers at the B3LYP/6-311++G(2d,p) level. (**a**) Regression analysis of calculated versus experimental ^13^C NMR chemical shifts of **1** and **2**. (**b**,**c**) Relative chemical shift errors between calculated and experimental ^13^C NMR data for the experimentally produced **1** and **2**.

Revolving around C-4 and C-7, *δ*_C_ values of four plausible isomers, **1A**, **1B**, **1C** and **1D** (Figure 3 and Figure 4), were calculated at the B3LYP/6-311++G(2d,p) level. The DP4+ probability analysis disclosed that isomer **1B** (4*R*, 7*R*) was the most likely one with a probability of 100%. Thus, the configuration of compound **1** was inferred as 3*S*,4*R*,7*R*,8*S*,9*S*,10*R*,11*R*,12*S*,15*S* on the basis of the GIAO NMR shift calculation followed by DP4 analysis.

Neo-debromoaplysiatoxin J (**2**) was isolated as a white solid. The molecular formula of C_32_H_46_O_11_ with 10 degrees of unsaturation was inferred from HRESIMS data at *m*/*z* 629.2938 [M + Na]^+^ (calcd. for C_32_H_46_O_11_Na, 629.2938). The NMR data of **2** exhibited a keto carbon (*δ*_C_ 207.2), two carbonyl carbons (*δ*_C_ 169.8 and 165.5), six aromatic carbons (*δ*_C_ 156.4, 143.5, 129.5, 118.4, 114.8 and 114.5), two oxygenated quaternary carbons (*δ*_C_ 105.7 and 87.2) and a methoxy (*δ*_C_ 56.7) (Table 1). The detailed interpretation of NMR spectra indicated that the planar structure of **2** closely resembles those of debromoaplysiatoxins, especially neo-debromoaplysiatoxin A (Neo-A) [10], except for C-2, C-4, C-7 and the atom composition of ring A, C. The HMBC correlations from H_2_-2 to C-1 and C-3; from H_3_-26 to C-3, C-4 and C-5; from H_2_-5 to C-3, C-4, C-6, C-7, C-25 and C-26; and from H_3_-24 and H_3_-25 to C-5, C-6 and C-7 decided the structural segment of C-1–C-7 (Figure 2). Considering the requirement of the unsaturation and the molecular formula for **2**, the C-4 (*δ*_C_ 87.2) and C-7 (*δ*_C_ 105.7) were linked through a peroxide bridge to form a relatively stable six-membered ring A. Besides, IR absorption bands for 877 cm^−1^ suggested a peroxide bond [24]. ^13^C chemical shifts of carbons in a similar chemical environment with C-4 and C-7 of **2**, such as C-3 (*δ*_C_ 105.4) of artemisinin [24], C-8 (*δ*_C_ 109.4) of hedychin A [25] and C-7 (*δ*_C_ 89.8) of talaperoxides B [26], verified the rationality of the chemical shifts attribution.

**Figure 5:**
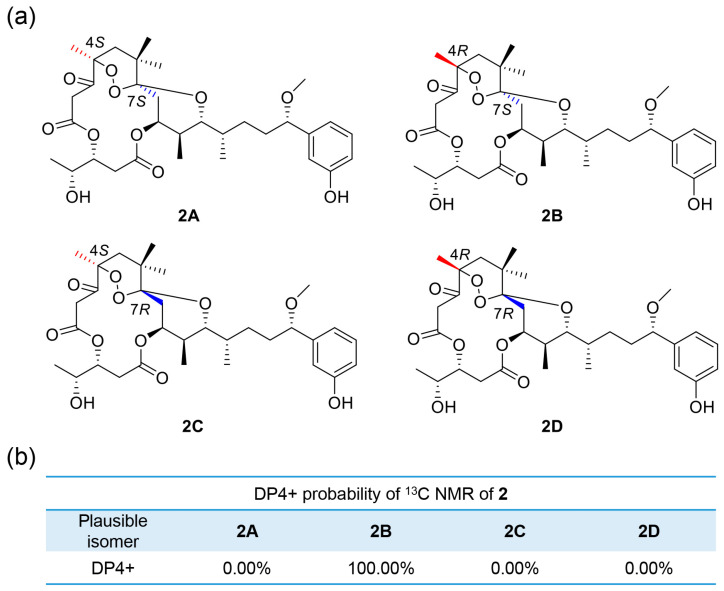
(**a**) Possible isomers for **2**. (**b**) DP4+ probability of ^13^C values of **2**.

The NOESY spectrum displayed correlations of H-15/H-13b and H-11/H-12/H_3_-23/H-13b, uncovering these protons shared *syn* relationships. H-11 was attached axially to the ring B according to the big coupling constants of H-11 (*J*_10, 11_= 10.7 Hz). The *syn* relationship of H-9 and H-10 was deduced from the NOE correlations of H-9/H-10/H_3_-22. H-9 presented an equatorial orientation with a small coupling constant (*J* = 2.8 Hz). H-5a exhibited a NOE correlation with H_3_-24, and H-5b had a NOE correlation with H_3_-24, H_3_-25 and H_3_-26, declaring H_3_-25 and H_3_-26 were on the same side of ring A. The common biosynthetic origin of ATXs and the coupling constant of H-29 and H-30 (*J*_29,30_ = 4.7 Hz), in accordance with existing ATXs [7,20,27], demonstrated C-29 and C-30 as 29*R*,30*R*. Considering *J*_11,12_ = 1.9 Hz, the relationship of H-11 and H-12 was determined the same way as **1**. The DP4+ analysis following the Gauge-Independent Atomic Orbital (GIAO) NMR shift calculation were performed (Figure 4), reflecting isomer **2B** (4*R*, 7*S*) (Figure 5) with a probability of 100% was the one fits the bill on the basis of the ^13^C NMR data. The configuration of compound **2** was settled as 4*R*,7*S*,9*S*,10*S*,11*R*,12*S*,15*S*,29*R*,30*R*.

**Scheme 1:**
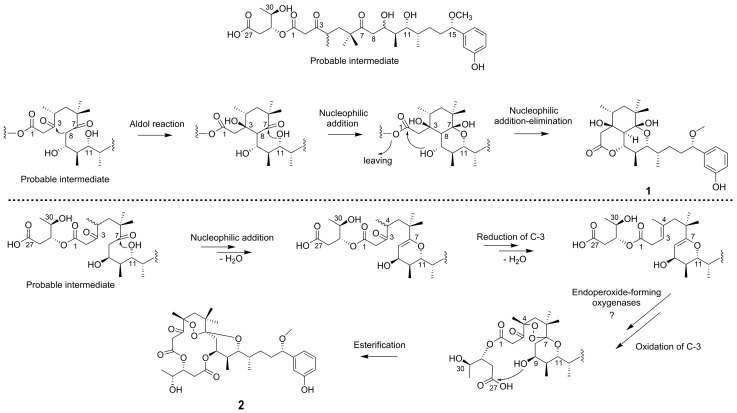
Postulated biosynthetic pathways of compounds **1** and **2**.

The two compounds are considered to have common biosynthetic intermediate [13,28], and their plausible biosynthesis pathway is proposed as shown in Scheme 1. Compound **1** is biosynthesized via aldol reaction between C-8 and C-3, followed by nucleophilic addition between 11-OH and C-7, generating 3-OH and 7-OH and ring A, B. Then, 9-OH acting as nucleophilic reagent undergoes transesterification with C-1 to give **1**. When it comes to compound **2**, nucleophilic addition between 11-OH/C-7 and dehydration of 7-OH/C-8 occurs first, followed by reduction of C-3 and dehydration of 3-OH/C-4. The introduction of peroxide bridge is proposed through endoperoxide-forming oxygenases after careful considerations and in combination with some findings published, such as the cyclooxygenases in the biosynthesis of prostaglandins [29] and fumitremorgin B endoperoxidase (FtmOx1) in the biosynthesis of verruculogen [30]. After oxidation of C-3, the esterification reaction of C-27/9-OH finally establishes compound **2**.

### 2.2. Bioactivities

#### 2.2.1. Inhibitory Activities against Kv1.5

**Figure 6:**
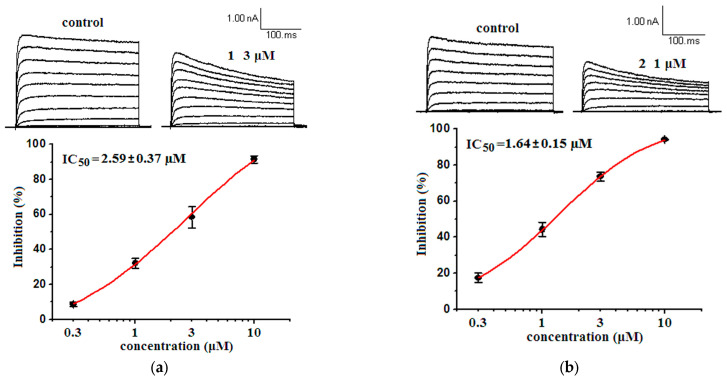
Kv1.5 currents and concentration–inhibition curve in the absence and presence of 0.3 µM, 1 µM, 3 µM and 10 µM compounds. The abscissa represents the concentration, and the ordinate represents the percentage of Kv1.5 current that is blocked at different concentrations of compounds. Data points represent mean ± SEM of 4 measurements. (**a**) The inhibitory effect of **1** showed an IC_50_ value of 2.59 ± 0.37 µM. (**b**) The inhibitory effect of **2** showed an IC_50_ value of 1.64 ± 0.15 µM.

The Kv1.5 K^+^ channel is considered as an effective and safe therapeutic target of atrial fibrillation because of its selective existence in atrium [31]. Considering the selective inhibitory effects on Kv1.5 of ATXs, we tested compounds **1** and **2** for their Kv1.5 inhibitory activities. The results showed **1** and **2** exhibited IC_50_ values of 2.59 ± 0.37 μM and 1.64 ± 0.15 μM, respectively (Figure 6). In all the ATXs tested for Kv1.5 inhibitory activities (Table S3), we preliminarily analyzed the relationship between different ATXs modified by different functional groups and Kv1.5 inhibitory activities (Figure S3). Generally speaking, oxygenated six-membered ring B is necessary for inhibition activities; for example, neo-debromoaplysiatoxin C without six-membered ring B exhibited no significant effect on the Kv1.5 channel. Ring C is not essential for the inhibition activities. Plus, configuration of chiral carbons also plays an important role, such as neo-debromoaplysiatoxin E and neo-debromoapysiatoxin F showing different inhibitory activities against Kv1.5. Different substituent groups on ring A of spiro structures also displayed a difference in activity.

#### 2.2.2. Cytotoxic Effects

Since the discovery of artemisinin, natural peroxides have attracted more and more attention. So far, a large number of polyketides and other chemical components with peroxy rings have been discovered from marine organisms [32,33]. Some of their bioactivities are attributed to the presence of endoperoxide. The endoperoxide bridge in artemisinin is essential for its antimalarial activity [34]. A sesquiterpene with an epidioxy bond bridge showed good anti-influenza virus activity, and the peroxy bridge may play a key role in increasing the activity [35]. In the report of Wibowo et al. [36], terpene endoperoxides exhibited cytotoxic activity, while those without an endoperoxide moiety did not show activity. The cytotoxic activity was probably caused by the reactive radical derived from a homolytic cleavage of endoperoxide.

**Figure 7:**
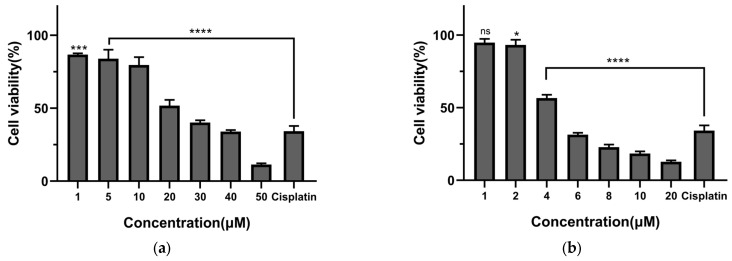
Cytotoxic effects of two compounds for 72 h on SW480 human colon cancer cells as measured using a cell viability assay. Cisplatin (5 μM) was used as positive control. Asterisks indicate statistical significance at *p* < 0.05 and ns means no significant differences, compared to the untreated control group. (**a**) SW480 exposed to different concentrations of **1**. (**b**) SW480 exposed to different concentrations of **2**. The data are expressed as the means ± SD of four independent experiments.

The intriguing structures of **1** and **2**, particularly the cyclic peroxide moiety, encouraged us to investigate their cytotoxic activities because many natural peroxides exhibited significant anticancer activities [37]. We assayed the cytotoxicity of two compounds toward the SW480 human colorectal cancer cell line. As expected, compound **2** exhibited obvious cytotoxicity with IC_50_ values of 4.63 ± 0.20 μM. Compound **1** showed relatively weak activity with IC_50_ values of 21.14 ± 2.20 μM (Figure 7 and Figure S1). In addition, cytotoxicity against 293A human kidney cells (human normal cell line), SGC7901 human gastric cancer cells, LoVo human colorectal carcinoma cells and PC-9 non-small-cell lung cancer cells were also tested (Figure S2). Similar with effects on SW480 cells, compound **1** mostly exhibited little or weak cytotoxicity against the three cancer cell lines. While compound **2** showed stronger cytotoxicity against the three cancer cells, with cell viability less than 20% at 20 μM. However, both compounds **1** and **2** exhibited no or less cytotoxicity against 293A human kidney cells from 1 μM to 20 μM.

Interestingly, among the previously reported cytotoxic tests against cancer cell lines like HeLa cells and L1210 mouse lymphoma cells [28,38,39], most existing ATXs exhibited weak or modest anticancer activities. It seems that debromoaplysiatoxin (IC_50_ values of 3.03 μM against HeLa cancer cells) [38] and neo-debromoaplysiatoxin J (**2**) possess relatively strong anticancer activities as natural sources of ATXs. **2** showing cell line-selective antiproliferative activities could be beneficial in cancer treatment and provided an interesting orientation in our following studies.

## 3. Materials and Methods

### 3.1. General Experimental Procedures

The UV spectrum was obtained using SPD-M20A diode array detector (Shimadzu, Kyoto, Japan). Optical rotation data were tested on an Autopol IV polarimeter (Rudolph, Hackettstown, NJ, USA). The FT-IR spectra were recorded using a Nicolet iS20 instrument (Thermo Fisher Scientific, Waltham, MA, USA), with a resolution of 4 cm^−1^ and 32 scans in % transmittance mode. A KBr-disk was used for preparing the samples. ^1^H and ^13^C NMR spectra were recorded in CDCl_3_ containing Me_4_Si as internal standard on Agilent 600 MHz spectrometer (Agilent Technologies, Santa Clara, CA, USA). HRESIMS was performed with a Bruker micrOTOF-Q II mass spectrometer (Bruker Daltonik GmbH, Bremen, Germany). HPLC was carried out on a Shimadzu LC-16 series instrument (Shimadzu, Kyoto, Japan), equipped with C-18 column (5 µm, 10 mm × 250 mm, SilGreen, Beijing, China) and an SPD-M20A diode array detector (Shimadzu, Kyoto, Japan). As for column chromatography, silica gel 60 (200–300 mesh; Yantai Jiangyou Silica Gel Development Co. LTD, Yantai, China) and octadecylsilyl (ODS) (50 μm, YMC, Kyoto, Japan) were used.

### 3.2. Material

The cyanobacterium sample was collected from Lingshui Port, Sanya, China, in November 2016. According to morphological and molecular identification, the sample belongs to *Lyngbya* sp. The voucher specimen numbered as BNH-201606 (gene bank accession numbers: MH636576) was deposited by Professor Bingnan Han in Zhejiang Sci-Tech University.

### 3.3. Extraction and Isolation

The freeze-dried cyanobacterium sample (150 g) was cut into pieces for ultrasonic extraction with CH_2_Cl_2_/MeOH (2:1, *v*/*v*). Then, the extract was suspended in 1 L of MeOH/H_2_O (3:2, *v*/*v*) and partitioned with CH_2_Cl_2_ (1 L) 3 times to yield the CH_2_Cl_2_ extract (20 g). Vacuum liquid chromatography (VLC) over silica gel was used for further separation to yield seven subfractions (F. A–G), with gradients of petroleum ether/EtOAc (5:1, 2:1, 1:1, 1:2, 1:5, 0:1, *v*/*v*). In which F.B (2000 mg) was subject to purification by reversed-phase octadecylsilyl silica (ODS) (UV detection at 220 nm, flow rate 20 mL/min, 10%–100% MeCN/H_2_O, 180 min), obtaining 17 subfractions (F.B.1–17). Next, F.B.7 (231mg) was isolated by semi-preparative HPLC (Shimadzu, C-18 (SilGreen), 70% MeCN/H_2_O, 2.0 mL/min, UV detection at 220 nm), and we acquired our two compounds **1** (6.3 mg, tR = 16.3 min) and **2** (3.2 mg, tR = 18.6 min).

*Neo-debromoaplysiatoxin I* (**1**): white solid; ${\left[\alpha \right]}_{D}^{22}$ +6.7 (c 0.03, MeOH); UV (MeCN) λ_max_ (log ε) 196 (3.27) nm, 276 (2.45) nm; IR (KBr) *ν*_max_: 3418 (br), 3260 (br), 2926, 1712, 1663, 1646, 1455, 1253, 1052, 919 cm^−1^; ^1^H NMR (600 MHz, CDCl_3_) and ^13^C NMR (150 MHz, CDCl_3_) data see Table 1; (+) HRESIMS *m/z* 499.2668 [M + Na]^+^ (calcd. for C_27_H_40_O_7_Na, 499.2672).

*Neo-debromoaplysiatoxin J* (**2**): white solid; ${\left[\alpha \right]}_{D}^{22}$ −35.7 (c 0.07, MeOH); UV (MeCN) λ_max_ (log ε) 196 (3.34) nm, 275 (2.56) nm; IR (KBr) *ν*_max_: 3418 (br), 2966, 2929, 1727, 1653, 1599, 1455, 1300, 1223, 1117, 1067, 1047, 877 cm^−1^; ^1^H NMR (600 MHz, CDCl_3_) and ^13^C NMR (150 MHz, CDCl_3_) data see Table 1; (+) HRESIMS *m*/*z* 629.2938 [M + Na]^+^ (calcd. for C_32_H_46_O_11_Na, 629.2938).

### 3.4. Theory and Calculation Details

The calculations, conformational search and optimization were performed as reported [12]. The stable conformations obtained at the B3LYP/6-31G(d) level were further used in magnetic shielding constants at the B3LYP/6-311++G(2d,p) level. The DP4+ calculations were finished with a simplified procedure according to Grimblat [40].

### 3.5. Cell Culture

The mouse connective tissue fibrocytes (LTK cells) stably expressing human Kv1.5 channels (LTK-Kv1.5) from Professor Weiping Wang at Chinese Academy of Medical Sciences and Peking Union Medical College, China, was cultured for ion channel inhibitory experiment. The 293A human kidney cell line, SW480 human colorectal cancer cell line, SGC7901 human gastric cancer cell line, LoVo human colorectal carcinoma cell line and PC-9 non-small-cell lung cancer cell line, used for cell viability assay, were kindly provided by Professor Yigang Wang and Professor Wenbin Ou at Zhejiang Sci-Tech University, China. Cells were grown in Dulbecco’s Modified Eagle Medium (DMEM) (Gibco, Shanghai, China), except PC-9 in RPMI-1640 medium (Gibco, Shanghai, China), containing 10% fetal bovine serum (FBS) (Wisent bioproducts, Montreal, Canada) and 1% antibiotics (10,000 U/mL penicillin, 10 mg /mL streptomycin) (Solarbio life sciences, Beijing, China) at 37 °C in a humidified atmosphere containing 5% CO_2_. When the cells grew to 70–85% confluence, they were passaged for subsequent tests. In all the tests, control cells were treated with DMSO (Solarbio Life Sciences, Beijing, China).

### 3.6. Ion Channel Inhibitory Experiment

The instruments and methods for the experiment mainly refer to previously reported [12]. Whole-oocyte recordings were conducted by electrode patch clamp. The intracellular fluid contained KCl (140 mM), MgCl_2_ (1 mM), HEPES (10 mM), Mg-ATP (3 mM) and EGTA (10 mM). The pH was adjusted to 7.25 by KOH. The extracellular fluid used to record currents contained NaCl (135 mM), KCl (5.4 mM), MgCl_2_ (1 mM), CaCl_2_ (1.8 mM), Glucose (10 mM), HEPES (10 mM) and NaH_2_PO_4_ (0.33 mM). pH was adjusted to 7.4 by NaOH.

Kv1.5 currents were evoked by a 300 ms depolarizing pulse from −50 mV to 50 mv in 10 mV increments from a holding potential of −70 mV. The current amplitude at the end of 300 ms pulse at 50 mV was measured. All values were indicated as mean ± SEM, and a value of *p* < 0.05 was considered to be significant.

### 3.7. Cell Viability Assay

The cells were seeded on 96-well plates (Nest, Wuxi, China) at a density of 3000 cells in 100 μL medium per well. After 16–20 h, the medium was changed into a fresh medium that had different concentrations of compounds. After a 72 h incubation period, 100 μL fresh medium with 10% CCK-8 reagent (Vazyme, Nanjing, China) was added along the hole-wall into each hole to replace the drug-containing medium. The plates were incubated for 1–2 h before the light absorption values at 450 nm of each group were detected. Cisplatin (5 μM) (Selleckchem, Houston, TX, USA), a kind of broad-spectrum anticancer drug for clinical application, was used as positive control in cell viability assay. Furthermore, the half inhibitory concentration (IC_50_) was calculated using GraphPad Prism (Version 8.0.2) software.

## 4. Conclusions

In conclusion, neo-debromoaplysiatoxin I (**1**), featuring a new skeleton, and neo-debromoaplysiatoxin J (**2**), with an unprecedented peroxide bridge group, were characterized from cyanobacterium *Lyngbya* sp. Two compounds all exhibited significant inhibitory activities against the Kv1.5 K^+^ channel with IC_50_ values of 2.59 ± 0.37 μM (**1**) and 1.64 ± 0.15 μM (**2**), respectively. Surprisingly, in the in vitro cytotoxic assays, **2** exhibited apparent stronger cytotoxic effects against four cancer cell lines, including SW480, SGC7901, LoVo and PC-9, than **1**, with cell viability less than 20% at 20 μM, while it exhibited no or less cytotoxicity against 293A human kidney cells (normal cells) from 1 μM to 20 μM.

## Figures and Tables

**Table 1:** ^1^H (600 MHz) and ^13^C (150 MHz) NMR data for compounds **1** and **2** in CDCl_3_ (*δ* in ppm).

Pos.	1		2	
	*δ*c, Type	*δ*_H_, m (*J* in Hz)	*δ*c, Type	*δ*_H_, m (*J* in Hz)
1	170.5, C		165.5, C	
2	40.9, CH_2_	a. 2.62, dd (17.7, 1.9)	46.0, CH_2_	a. 4.00, d (10.8)
		b. 2.55, d (17.7)		b. 3.26, d (10.7)
3	72.6, C		207.2, C	
4	38.0, CH	1.96, overlap	87.2, C	
5	40.1, CH_2_	a. 2.02, dd (14.4, 5.8)	40.2, CH_2_	a. 2.27, d (13.8)
		b. 1.21, dd (14.6, 2.1)		b. 1.44, d (13.8)
6	39.5, C		39.0, C	
7	100.1, C		105.7, C	
8	40.2, CH	1.96, overlap	29.4, CH_2_	a. 2.96, dd (14.8, 3.2)
				b. 1.46, dd (14.8, 2.6)
9	77.6, CH	4.54, t (10.7)	74.2, CH	4.71, q (2.8)
10	39.3, CH	1.78, m	34.4, CH	1.63, overlap
11	75.5, CH	3.55, dd (10.4, 1.9)	74.1, CH	3.61, dd (10.7, 1.9)
12	33.0, CH	1.71, hept (6.1)	33.7, CH	1.28, overlap
13	30.1, CH_2_	a. 1.36, m	29.9, CH_2_	a. 1.25, overlap
		b. 1.25, overlap		b. 1.21, m
14	35.8, CH_2_	a. 1.83, m	35.2, CH_2_	a. 1.81, m
		b. 1.62, overlap		b. 1.65, overlap
15	84.2, CH	4.01, t (6.6)	85.0, CH	4.04, dd (8.5, 5.2)
16	144.4, C		143.6, C	
17	119.5, CH	6.82, dt (7.5, 1.2)	118.4, CH	6.85, overlap
18	129.8, CH	7.20, t (7.8)	129.5, CH	7.20, t (8.0)
19	114.8, CH	6.74, ddd (8.1, 2.6, 1.0)	114.8, CH	6.78, dt (8.4, 1.4)
20	156.0, C		156.4, C	
21	113.5, CH	6.77, t (2.0)	114.5, CH	6.85, overlap
22	13.1, CH_3_	0.82, d (6.6)	12.0, CH_3_	0.71, d (6.6)
23	12.3, CH_3_	1.01, d (6.4)	13.6, CH_3_	0.74, d (6.9)
24	23.4, CH_3_	0.83, s	25.6, CH_3_	0.85, s
25	24.8, CH_3_	1.06, s	26.2, CH_3_	1.12, s
26	19.7, CH_3_	1.08, d (7.7)	22.8, CH_3_	1.28, s
27	56.8, CH_3_	3.20, s	169.8, C	
28			35.2, CH_2_	a. 2.79, dd (16.9, 1.5)
				b. 2.70, dd (16.9, 10.9)
29			75.0, CH	5.33, dd (10.7, 4.7)
30			67.4, CH	3.95, m
31			17.4, CH_3_	1.15, d (6.4)
32			56.7, CH_3_	3.25, s
3-OH		4.35, d (2.0)		

## Data Availability

Data are contained within the article or Supplementary Materials.

## Supplementary Materials

The following supporting information can be downloaded at: https://www.mdpi.com/article/10.3390/molecules28062786/s1, Tables S1 and S2: Detailed NMR data of compounds **1** and **2**; Table S3: Kv1.5 inhibitory effects of ATXs; Tables S4 and S5: The calculated ^13^C NMR data for isomers of compounds **1** and **2**; Tables S6–S14: DFT-optimized structures and thermodynamic parameters for low-energy conformers of **1A**–**1D** and **2A**–**2D**; Figure S1: Dose-dependent effects of **1** and **2** on SW480 cell survival rate (72 h); Figure S2: Cytotoxic effects of two compounds for 72 h on different cells as measured using a cell viability assay; Figure S3: Critical structural features of ATXs leading to differential Kv1.5 inhibitory activities; Figures S4–S26: UV, IR, HR-ESI-MS, 1D NMR and 2D NMR spectra of compounds **1** and **2**; Figure S27: Morphological and molecular identification of cyanobacterium.
