# Supplementary material for: Structure Elucidation of Two Intriguing Neo-Debromoaplysiatoxin Derivatives from Marine Cyanobacterium Lyngbya sp. Showing Strong Inhibition of Kv1.5 Potassium Channel and Differential Cytotoxicity
Source: Molecules. 2023 Mar 20;28(6):2786. doi: 10.3390/molecules28062786 (PMC10059712; doi:10.3390/molecules28062786)
Supplement: Supplementary file 1 [file molecules-28-02786-s001.zip › molecules-2256513-supplementary.pdf]

Supporting Information

Structure elucidation of two intriguing neo-debromoaplysiatoxin derivatives from marine cyanobacterium *Lyngbya* sp. showing strong inhibition of Kv1.5 potassium channel and differential cytotoxicity

Zijun Chen [1], Na Chen [1], Peng Fu [2], Weiping Wang [3], Shilin Bian [1], Huihui Zhang [1], Sicheng Shen [1], Bingnan Han [1,*]

[1] Department of Development Technology of Marine Resources, College of Life Sciences and Medicine, Zhejiang Sci-Tech University, Hangzhou, 310018, China

[2] Key Laboratory of Marine Drugs, Ministry of Education of China, School of Medicine and Pharmacy, Ocean University of China, Qingdao, 266003, China

[3] Institute of Materia Medica, Chinese Academy of Medical Sciences and Peking Union Medical College, Beijing, 100730, China

* Corresponding author E-mail address: hanbingnan@zstu.edu.cn

Contents

Table S1. Detailed NMR data of compound **1** ···· 4

Table S2. Detailed NMR data of compound **2** ···· 4

Table S3. Kv1.5 inhibitory effects of ATXs ···· 6

Table S4. The calculated ^{13}C NMR data for isomers of compound **1** ···· 10

Table S5. The calculated ^{13}C NMR data for isomers of compound **2** ···· 11

Table S6. DFT-optimized structures and thermodynamic parameters for low-energy conformers of **1A** ···· 12

Table S7. DFT-optimized structures and thermodynamic parameters for low-energy conformers of **1B** ···· 13

Table S8. DFT-optimized structures and thermodynamic parameters for low-energy conformers of **1C** ···· 13

Table S9. DFT-optimized structures and thermodynamic parameters for low-energy conformers of **1D** ···· 14

Table S10. DFT-optimized structures and thermodynamic parameters for low-energy conformers of **2A** ···· 15

Table S11. DFT-optimized structures and thermodynamic parameters for low-energy conformers of **2B** ···· 16

Table S12. DFT-optimized structures and thermodynamic parameters for low-energy conformers of **2B** ···· 17

Table S13. DFT-optimized structures and thermodynamic parameters for low-energy conformers of **2C** ···· 18

Table S14. DFT-optimized structures and thermodynamic parameters for low-energy conformers of **2D** ···· 19

Figure S1. Dose-dependent effects of **1** and **2** on SW480 cell survival rate (72h).···· 20

Figure S2. Cytotoxic effects of two compounds for 72 h on different cells as measured using a cell viability assay.···· 21

Figure S3. Critical structural features of ATXs leading to differential Kv1.5 inhibitory activities.···· 22

Figure S4. ^{1}H NMR spectrum of Compound **1** (600 MHz, $CDCl_3$) ···· 23

Figure S5. ^{13}C NMR spectrum of Compound **1** (150 MHz, $CDCl_3$) ···· 24

Figure S6. DEPT spectrum of Compound **1** (150 MHz, $CDCl_3$) ···· 25

Figure S7. HSQC spectrum of Compound **1** (600 MHz, $CDCl_3$)···· 26

Figure S8. ^{1}H-^{1}H COSY spectrum of Compound **1** (600 MHz, $CDCl_3$)···· 27

Figure S9. Expansion of ^{1}H-^{1}H COSY spectrum of Compound **1** (600 MHz, $CDCl_3$) ······· 28

Figure S10. Expansion of ^{1}H-^{1}H COSY spectrum of Compound **1** (600 MHz, $CDCl_3$)······ 29

Figure S11. HMBC spectrum of Compound **1** (600 MHz, $CDCl_3$) ···························· 30

Figure S12. NOESY spectrum of Compound **1** (600 MHz, $CDCl_3$) ··························· 31

Figure S13. HRESIMS spectrum of Compound **1** in MeOH ···································· 32

Figure S14. UV spectrum of Compound **1** in MeCN ··· 32

Figure S15. IR spectrum of Compound **1** ··· 33

Figure S16. ^{1}H NMR spectrum of Compound **2** (600 MHz, $CDCl_3$) ·························· 34

Figure S17. ^{13}C NMR spectrum of Compound **2** (150 MHz, $CDCl_3$)························· 35

Figure S18. DEPT spectrum of Compound **2** (150 MHz, $CDCl_3$)······························ 36

Figure S19. HSQC spectrum of Compound **2** (600 MHz, $CDCl_3$) ····························· 37

Figure S20. ^{1}H-^{1}H COSY spectrum of Compound **2** (600 MHz, $CDCl_3$) ····················· 38

Figure S21. Expansion of ^{1}H-^{1}H COSY spectrum of Compound **2** (600 MHz, $CDCl_3$)······ 39

Figure S22. HMBC spectrum of Compound **2** (600 MHz, $CDCl_3$) ···························· 40

Figure S23. NOESY spectrum of Compound **2** (600 MHz, $CDCl_3$) ··························· 41

Figure S24. HRESIMS spectrum of Compound **2** in MeOH ···································· 42

Figure S25. UV spectrum of Compound **2** in MeCN ··· 42

Figure S26. IR spectrum of Compound **2** ··· 43

Figure S27. Morphological and molecular identification of cyanobacterium················· 45

1. Tables

Table S1. Detailed NMR data of compound **1**

Pos.	**1**				
	δ_C, type	δ_H, m (*J* in Hz)	HMBC	COSY	NOESY
1	170.5, C				
2	40.9, CH_2	a. 2.62, dd (17.7, 1.9)	C-1		H-26
		b. 2.55, d (17.7)	C-1, 3, 8		H-26
3	72.6, C				
4	38.0, CH	1.96, overlap	C-3, 5, 6, 8, 26	H-5a, 26	H-2, 5b
5	40.1, CH_2	a. 2.02, dd (14.4, 5.8)	C-4, 6, 25, 26	H-4, 5b	H-24
		b. 1.21, dd (14.6, 2.1)	C-3, 4, 6, 7, 26	H-5a	H-4, 5a, 24, 25, 26
6	39.5, C				
7	100.1, C				
8	40.2, CH	1.96, overlap	C-3, 7, 9, 10	H-9	H-2, 10
9	77.6, CH	4.54, t (10.7)		H-8, 10	H-11, 23
10	39.3, CH	1.78, m	C-11	H-9, 11, 23	H-8, 23, 22
11	75.5, CH	3.55, dd (10.4, 1.9)	C-22	H-10, 12	H-9, 23
12	33.0, CH	1.71, hept (6.1)	C-13, 22	H-11, 22	H-11, 23
13	30.1, CH_2	a. 1.36, m	C-12	H-12	H-12, 15, 22
		b. 1.25, overlap			
14	35.8, CH_2	a. 1.83, m	C-15, 16	H-15	H-12, 15
		b. 1.62, overlap		H-15	
15	84.2, CH	4.01, t (6.6)	C-13, 14, 17, 21, 27	H-14ab	H-17, 21, 27
16	144.4, C				
17	119.5, CH	6.82, dt (7.5, 1.2)	C-19	H-18	H-15
18	129.6, CH	7.20, t (7.8)	C-16, 20	H-17, 19	H-17, 19
19	114.8, CH	6.74, ddd (8.1, 2.6, 1.0)	C-17, 21	H-18	H-18
20	156.0, C				
21	113.5, CH	6.77, t (2.0)	C-17, 19	H-17	H-15
22	13.1, CH_3	0.82, d (6.6)	C-11, 12, 13	H-12	H-10, 12
23	12.3, CH_3	1.01, d (6.4)	C-9, 10, 11	H-10	H-9, 11
24	23.4, CH_3	0.83, s	C-5, 6, 7, 25	H-25	H-5a, 25
25	24.8, CH_3	1.06, s	C-5, 6, 7, 24	H-24	H24
26	19.7, CH_3	1.08, d (7.7)	C-3, 4, 5, 8	H-4, 5a	H-2, 5b
27	56.8, CH_3	3.20, s	C-15		H-15
3-OH		4.35, d (2.0)	C-2	H-2a	H-5a, 9

Table S2. Detailed NMR data of compound **2**

Pos.	**2**

	δc, type	δ_H, m (*J* in Hz)	HMBC	COSY	NOESY
1	165.5, C				
2	46.0, CH_2	a. 4.00, d (10.8)	C-1, 3	H-2b	H-2b, 26
		b. 3.26, d (10.7)	C-1, 3, 4	H-2a	
3	207.2, C				
4	87.2, C				
5	40.2, CH_2	a. 2.27, d (13.8)	C-3, 4, 6, 7, 25, 26	H-5b	H-5b, 24
		b. 1.44, d (13.8)	C-3, 4, 6, 7, 25, 26	H-5a	H-24, 25, 26
6	39.0, C				
7	105.7, C				
8	29.4, CH_2	a. 2.96, dd (14.8, 3.2)	C-7, 9, 10		H-8b, 9, 25
		b. 1.46, dd (14.8, 2.6)			H-9, 24, 25
9	74.2, CH	4.71, q (2.8)	C-7, 11	H-8ab, 10	H-8ab, 10, 23
10	34.4, CH	1.63, overlap	C-9, 11, 23	H-23	H-22
11	74.1, CH	3.61, dd (10.7, 1.9)	C-9, 12, 13, 22	H-10, 12	H-12, 13b, 23, 29
12	33.7, CH	1.28, overlap	C-13, 22	H-22	H-11, 23
13	29.9, CH_2	a. 1.25, overlap	C-15		H-22
		b. 1.21, m	C-11, 12, 15		H-22
14	35.2, CH_2	a. 1.81, m	C-12, 13, 15, 16	H-13ab,14b	H-13ab, 14b
		b. 1.65, overlap	C-13, 15	H-13ab	
15	85.0, CH	4.04, dd (8.5, 5.2)	C-13, 14, 17, 21, 32	H-14ab	H-13b, H14a, 32
16	143.6, C				
17	118.4, CH	6.85, overlap	C-15, 18, 19, 21	H-18, 19	H-15, 18, 32
18	129.5, CH	7.20, t (8.0)	C-16, 19, 20	H-17, 19	H-17, 19
19	114.8, CH	6.78, dt (8.4, 1.4)	C-17, 20, 21	H-18	H-18
20	156.4, C				
21	114.5, CH	6.85, overlap	C-15, 17, 19, 20		
22	12.0, CH_3	0.71, d (6.6)	C-11, 12, 13		
23	13.6, CH_3	0.74, d (6.9)	C-9, 10, 11		
24	25.6, CH_3	0.85,s	C-4, 5, 6, 7, 25		H-22
25	26.2, CH_3	1.12, s	C-5, 6, 7, 24	H-24	H-24, 26
26	22.8, CH_3	1.28, s	C-3, 4, 5		
27	169.8, C				
28	35.2, CH_2	a. 2.79, dd (16.9, 1.5)	C-27, 29, 30		H-31
		b. 2.70, dd (16.9, 10.9)	C-27, 29		H-31
29	75.0, CH	5.33, dd (10.7, 4.7)		H-28, 30	H-11, 28a, 30
30	67.4, CH	3.95, m	C-28, 29, 31	H-31	H-28a, 31
31	17.4, CH_3	1.15, d (6.4)	C29,30		
32	56.7, CH_3	3.25, s	C15		

Table S3. Kv1.5 inhibitory effects of ATXs

No.	Name	Model	Inhibiting KV1.5 IC_{50} /μM	Reference
1	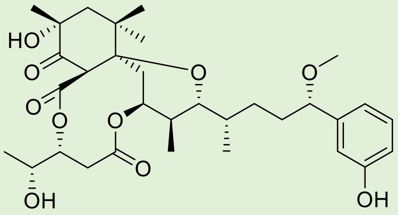 Neo-debromoaplysiatoxin A	CHO cells	6.94 ± 0.26	[10]
2	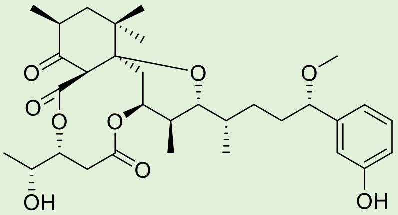 Neo-debromoaplysiatoxin D	CHO cells	1 μM with 22.4% inhibition	[20]
3	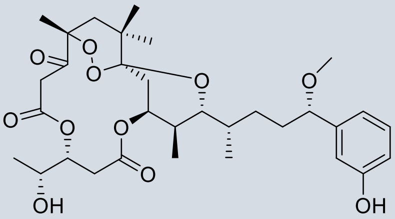 Neo-debromoaplysiatoxin J	LTK cells	1.64 ± 0.15	
4	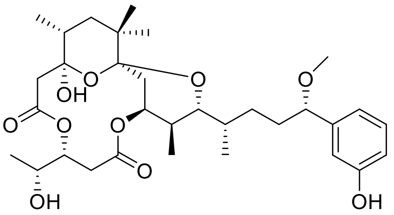 Debromoaplysiatoxin	CHO cells	1.28 ± 0.08<br>1 μM with 48.68% inhibition	[20]
5	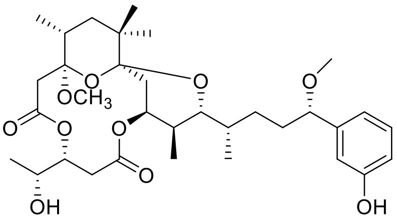 3-methoxydebromoaplysiatoxin	CHO cells	1 μM with 48.81% inhibition	[20]

6	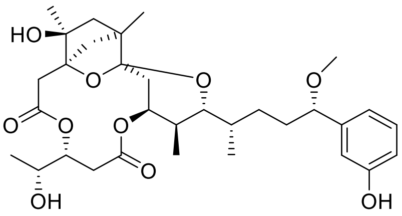<br>Neo-debromoaplysiatoxin G	CHO cells	1.79 ± 0.22	[11]
7	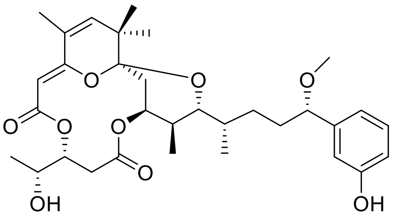<br>Oscillatoxin J	LTK cells	2.61 ± 0.91	[12]
8	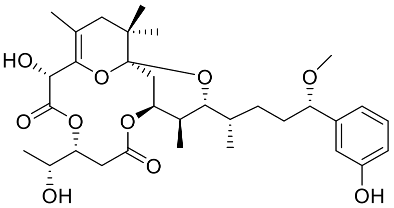<br>Oscillatoxin K	LTK cells	3.86 ± 1.03	[12]
9	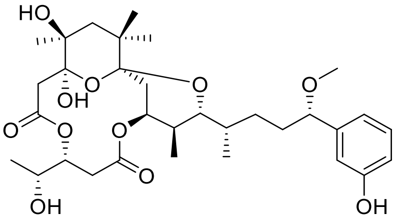<br>Oscillatoxin L	LTK cells	10 μM with minimum effect	[12]
10	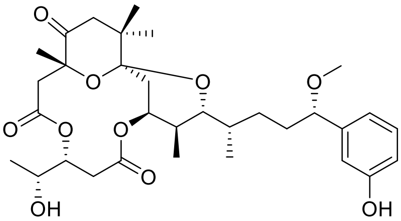<br>Oscillatoxin M	LTK cells	3.79 ± 1.01	[12]
11	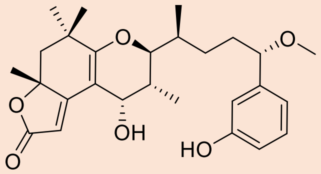<br>Neo-debromoaplysiatoxin E	CHO cells	1.22 ± 0.22	[21]

12	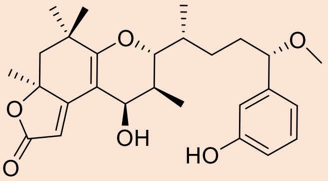<br>Neo-debromoaplysiatoxin F	CHO cells	2.85 ± 0.29	[21]
13	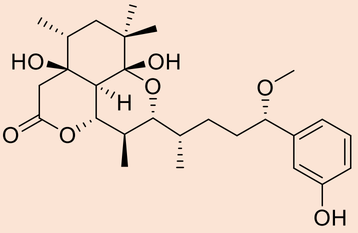<br>Neo-debromoaplysiatoxin I	LTK cells	2.59 ± 0.37	
14	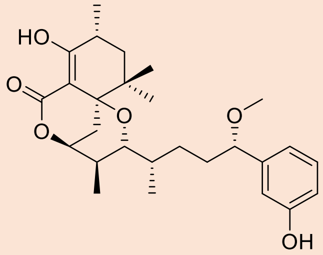<br>Neo-debromoaplysiatoxin B	CHO cells	0.30 ± 0.05	[10]
15	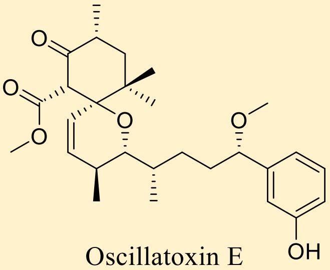<br>Oscillatoxin E	CHO cells	0.79 ± 0.032<br>1 μM with<br>65.40%<br>inhibition	[20]
16	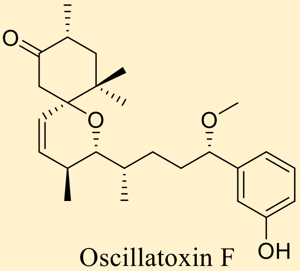<br>Oscillatoxin F	CHO cells	1 μM with<br>29.1%<br>inhibition	[20]
17	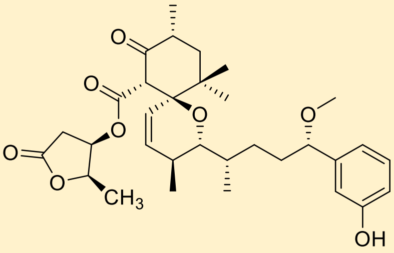<br>30-methyloscillatoxin D	CHO cells	1.47 ± 0.138<br>1 μM with<br>43.36%<br>inhibition	[20]

18	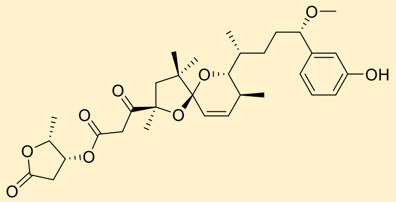 Neo-debromoaplysiatoxin H	CHO cells	1.46 ± 0.14	[11]
19	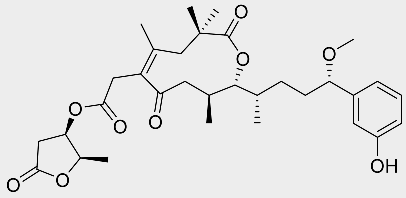 Neo-debromoaplysiatoxin C	CHO cells	no significant effect	[22]

Table S4. The calculated ^{13}C NMR data for isomers of compound **1**

No.	δexp	δcal				δscal				corrected error			
		**1A**	**1B**	**1C**	**1D**	**1A**	**1B**	**1C**	**1D**	**1A**	**1B**	**1C**	**1D**
1	170.5	180.732	174.8293	173.8694	177.6234	173.1473	167.306	166.9093	169.3056	2.647338	-3.19396	-3.59072	-1.19438
2	40.9	50.93348	44.44781	50.7826	46.91496	45.25439	40.70979	46.11756	43.0417	4.354393	-0.19021	5.217563	2.141697
3	72.6	77.99977	79.37732	76.83491	76.12895	71.92331	74.62523	71.68411	71.26231	-0.67669	2.025226	-0.91589	-1.33769
4	38	45.24957	46.05997	39.53414	43.19416	39.65392	42.27515	35.07885	39.44741	1.653922	4.275145	-2.92115	1.447411
5	40.1	45.03235	44.37029	47.1275	46.33567	39.4399	40.63452	42.53062	42.4821	-0.6601	0.534522	2.430622	2.382102
6	39.5	45.76417	45.93594	46.9748	46.61741	40.16097	42.15472	42.38076	42.75426	0.66097	2.654718	2.880761	3.25426
7	100.1	107.1988	108.0057	106.7185	107.7441	100.6937	102.4225	101.0105	101.8025	0.593695	2.322489	0.910523	1.702465
8	40.2	49.42871	44.93617	57.91087	48.66954	43.77171	41.18397	53.11293	44.73661	3.571711	0.983967	12.91293	4.53661
9	77.6	84.28008	81.04735	82.15292	80.6129	78.11142	76.24677	76.90296	75.5938	0.511419	-1.35323	-0.69704	-2.0062
10	39.3	44.1562	45.17277	43.77915	43.23725	38.57661	41.4137	39.2447	39.48903	-0.72339	2.113699	-0.0553	0.189033
11	75.5	91.08365	78.20164	83.65602	82.2514	84.8151	73.48368	78.37804	77.17659	9.315104	-2.01632	2.878037	1.676587
12	33	43.25647	39.01858	40.95819	41.29083	37.69009	35.43818	36.47634	37.6088	4.690086	2.438175	3.476343	4.608802
13	30.1	31.95872	30.97142	33.22308	30.74578	26.5582	27.62464	28.88546	27.42231	-3.5418	-2.47536	-1.21454	-2.67769
14	35.8	37.14285	40.6145	42.48027	41.22721	31.66622	36.98776	37.97003	37.54734	-4.13378	1.187762	2.170034	1.747339
15	84.2	90.00734	89.55207	91.35045	89.40725	83.7546	84.50458	85.929	84.08911	-0.4454	0.304585	1.729003	-0.11089
16	144.4	150.0582	154.8721	153.1004	153.855	142.9239	147.9283	146.5276	146.3454	-1.47612	3.528285	2.127573	1.945406
17	119.5	122.4299	126.0848	124.8747	125.6959	115.7012	119.9767	118.8282	119.1439	-3.79882	0.476702	-0.6718	-0.35612
18	129.6	135.0576	134.8567	134.241	134.3591	128.1435	128.4939	128.0198	127.5125	-1.45655	-1.10608	-1.5802	-2.08752
19	114.8	118.1933	118.5235	118.0608	119.9872	111.5267	112.6349	112.1413	113.6293	-3.27327	-2.16512	-2.65872	-1.17072
20	156	165.366	164.0546	165.3753	165.7425	158.0069	156.8441	158.5736	157.8288	2.006879	0.844127	2.573633	1.828758
21	113.5	119.0849	119.2604	117.0608	118.2847	112.4053	113.3505	111.16	111.9847	-1.09471	-0.14954	-2.34005	-1.51534
22	13.1	17.1195	15.60403	15.19788	19.92994	11.93684	12.7034	11.19635	16.97425	-1.16316	-0.3966	-1.90365	3.874247
23	12.3	21.10206	13.4682	11.62456	14.17169	15.86093	10.62958	7.689658	11.4118	3.560929	-1.67042	-4.61034	-0.8882
24	23.4	28.91663	24.35306	27.3613	23.93502	23.56077	21.19843	23.13297	20.84314	0.160773	-2.20157	-0.26703	-2.55686
25	24.8	28.17529	26.14444	25.75869	23.53791	22.83032	22.9378	21.56025	20.45953	-1.96968	-1.8622	-3.23975	-4.34047
26	19.7	20.2326	21.13076	14.88594	14.61379	15.00424	18.06968	10.89022	11.83886	-4.69576	-1.63032	-8.80978	-7.86114
27	56.8	58.9637	57.71339	57.78892	57.87025	53.16672	53.59024	52.99324	53.62447	-3.63328	-3.20976	-3.80676	-3.17553

Table S5. The calculated ^{13}C NMR data for isomers of compound **2**

No.	δexp	δcal				δscal				corrected error			
		**2A**	**2B**	**2C**	**2D**	**2A**	**2B**	**2C**	**2D**	**2A**	**2B**	**2C**	**2D**
1	165.5	181.4001	177.5256	178.3141	179.5045	171.3943	168.853	168.5209	167.6039	5.894292	3.353043	3.020907	2.103927
2	46	52.61359	52.21284	52.57882	49.45896	47.72757	48.22077	47.0962	45.23117	1.72757	2.22077	1.096202	-0.76883
3	207.2	220.0939	216.8758	219.4245	222.0823	208.5498	206.7334	208.2219	207.6697	1.34979	-0.46657	1.021888	0.469655
4	87.2	94.96897	94.23802	95.6802	94.75816	88.39915	88.67628	88.71994	87.85769	1.199148	1.476284	1.519939	0.657687
5	40.2	46.01818	46.04717	48.3404	44.63408	41.39435	42.28539	43.00309	40.69095	1.194352	2.085393	2.803092	0.490954
6	39	43.90874	45.9637	46.58448	44.86829	39.36877	42.20504	41.30736	40.91135	0.368772	3.20504	2.307365	1.911347
7	105.7	111.666	111.7316	113.5406	117.1514	104.4324	105.5165	105.968	108.9297	-1.26762	-0.1835	0.267994	3.229695
8	29.4	31.79937	32.95649	24.5162	35.74482	27.7408	29.68366	19.99565	32.32617	-1.6592	0.283659	-9.40435	2.92617
9	74.2	80.1222	80.91394	78.9369	80.44187	74.1426	75.84987	72.55065	74.38606	-0.0574	1.649869	-1.64935	0.186061
10	34.4	41.5062	39.58155	35.62803	37.18249	37.06174	36.06127	30.72654	33.67901	2.661741	1.661271	-3.67346	-0.72099
11	74.1	74.41987	80.1489	91.80512	93.5076	68.66696	75.1134	84.97771	86.68091	-5.43304	1.013397	10.87771	12.58091
12	33.7	39.7327	39.45948	45.48051	39.91803	35.35875	35.94376	40.24125	36.25315	1.658748	2.243765	6.541249	2.553153
13	29.9	34.47263	32.57743	30.77812	26.80129	30.30779	29.31876	26.04289	23.91031	0.407791	-0.58124	-3.85711	-5.98969
14	35.2	43.87786	39.58539	42.16241	32.57128	39.33912	36.06497	37.0369	29.33987	4.139116	0.864965	1.836903	-5.86013
15	85	90.79828	91.33894	90.00142	87.22318	84.39426	85.88548	83.23585	80.76727	-0.60574	0.885481	-1.76415	-4.23273
16	143.6	151.569	152.2225	151.7256	151.7255	142.7491	144.495	142.8439	141.4639	-0.85092	0.894996	-0.75608	-2.13607
17	118.4	122.0869	121.515	125.6206	124.6129	114.439	114.9344	117.6339	115.951	-3.96096	-3.4656	-0.76606	-2.44904
18	129.5	134.0495	134.6738	134.3758	135.0864	125.926	127.6017	126.0889	125.8065	-3.57398	-1.89826	-3.41107	-3.69349
19	114.8	120.8491	117.422	120.9268	120.6498	113.2504	110.9943	113.101	112.2217	-1.54958	-3.80573	-1.69901	-2.57826
20	156.4	168.7066	164.241	167.0414	165.8402	159.2054	156.0646	157.6347	154.7459	2.80536	-0.33536	1.234696	-1.65413
21	114.5	115.9907	120.3778	115.681	119.6786	108.5852	113.8397	108.035	111.3078	-5.91478	-0.66026	-6.46496	-3.1922
22	12	15.53627	11.91395	17.68642	18.47009	12.12423	9.427075	13.40002	16.07066	0.124231	-2.57292	1.40002	4.070662
23	13.6	15.68173	14.12012	20.89024	12.57325	12.26391	11.55084	16.49401	10.52173	-1.33609	-2.04916	2.894007	-3.07827
24	25.6	27.32353	25.40607	27.68932	26.81902	23.44289	22.41526	23.05999	23.92699	-2.15711	-3.18474	-2.54001	-1.67301
25	26.2	25.64804	27.30224	25.56872	28.0184	21.83401	24.24061	21.01209	25.05561	-4.36599	-1.95939	-5.18791	-1.14439
26	22.8	26.59061	25.25386	27.00492	25.86137	22.73912	22.26873	22.39906	23.02585	-0.06088	-0.53127	-0.40094	0.225851
27	169.8	177.3844	177.0999	178.5945	185.8451	167.5382	168.4432	168.7917	173.5705	-2.26175	-1.35683	-1.00834	3.77048
28	35.2	42.69638	42.06989	43.33485	40.56047	38.20461	38.45667	38.16915	36.85769	3.004611	3.256673	2.969149	1.657688

29	75	92.79356	81.56939	82.75867	82.94956	86.31022	76.48083	76.2414	76.7458	11.31022	1.480832	1.241401	1.745798
30	67.4	76.09364	75.98393	78.54966	74.92332	70.27419	71.104	72.17669	69.19311	2.874192	3.703996	4.776687	1.79311
31	17.4	19.81213	16.20361	23.42518	21.90672	16.2301	13.55652	18.94204	19.30453	-1.1699	-3.84348	1.542039	1.904529
32	56.7	57.20582	57.41619	57.47277	58.39959	52.13724	53.22977	51.82237	53.64429	-4.56276	-3.47023	-4.87763	-3.05571

Table S6. DFT-optimized structures and thermodynamic parameters for low-energy conformers of **1A**

Conformers	Conf. A	Conf. B	Conf. C	Conf. D	Conf. E
DFT-optimized structures	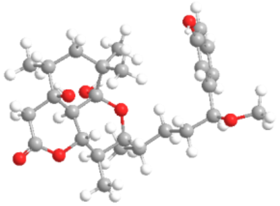	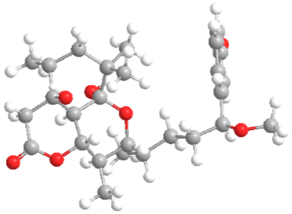	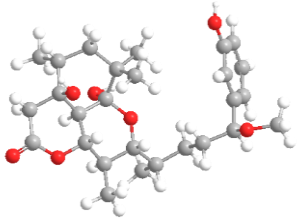	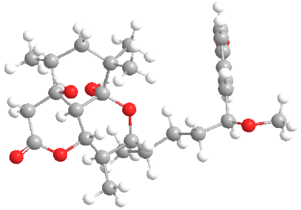	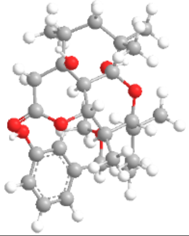
Population	0.94%	0.32%	0.17%	0.30%	98.28%
Total energy (a.u.)	-1579.50542388	-1579.50442238	-1579.50381390	-1579.50434043	-1579.50981307
Sum of electronic and zero-point energies (a.u.)	-1578.861355	-1578.860333	-1578.860134	-1578.860236	-1578.864071
Sum of electronic and thermal energies (a.u.)	-1578.826909	-1578.825931	-1578.825521	-1578.825840	-1578.830552
Sum of electronic and thermal enthalpies (a.u.)	-1578.825964	-1578.824987	-1578.824577	-1578.824895	-1578.829608
Sum of electronic and thermal free energies (a.u.)	-1578.927159	-1578.926329	-1578.926106	-1578.926176	-1578.925130

Table S7. DFT-optimized structures and thermodynamic parameters for low-energy conformers of **1B**

Conformers	Conf. A	Conf. B	Conf. C	Conf. D
DFT-optimized structures	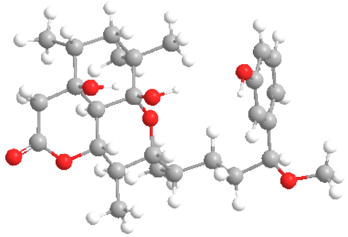	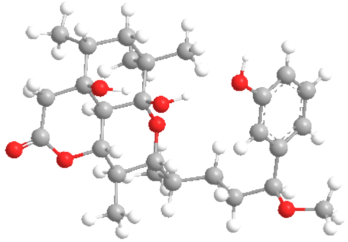	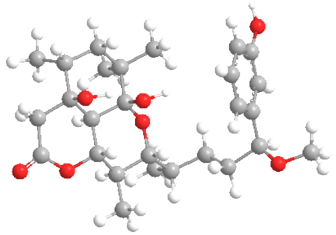	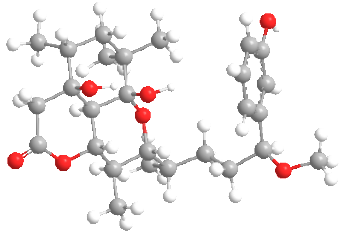
Population	25.17%	33.17%	20.96%	20.16%
Total energy (a.u.)	-1579.53074733	-1579.53102256	-1579.53057441	-1579.53053814
Sum of electronic and zero-point energies (a.u.)	-1578.885912	-1578.885465	-1578.885225	-1578.885145
Sum of electronic and thermal energies (a.u.)	-1578.851891	-1578.851850	-1578.851404	-1578.851337
Sum of electronic and thermal enthalpies (a.u.)	-1578.850947	-1578.850906	-1578.850460	-1578.850393
Sum of electronic and thermal free energies (a.u.)	-1578.950988	-1578.948290	-1578.949003	-1578.948944

Table S8. DFT-optimized structures and thermodynamic parameters for low-energy conformers of **1C**

Conformers	Conf. A	Conf. B	Conf. C	Conf. D
DFT-optimized structures	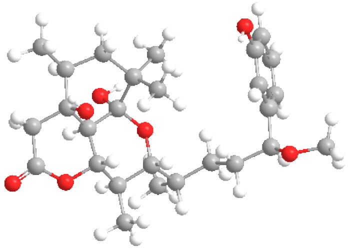	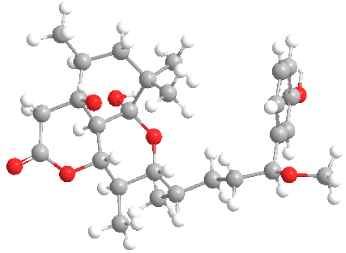	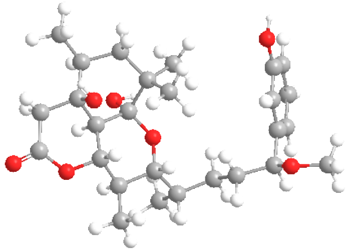	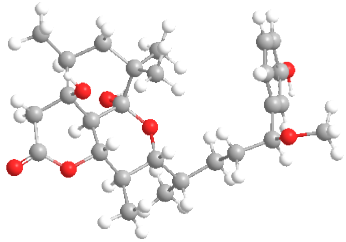
Population	52.37%	14.12%	20.00%	13.51%

Total energy (a.u.)	-1579.50954950	-1579.50831352	-1579.50864160	-1579.50827193
Sum of electronic and zero-point energies (a.u.)	-1578.865864	-1578.864781	-1578.865274	-1578.864687
Sum of electronic and thermal energies (a.u.)	-1578.831344	-1578.830176	-1578.830634	-1578.830105
Sum of electronic and thermal enthalpies (a.u.)	-1578.830399	-1578.829232	-1578.829690	-1578.829160
Sum of electronic and thermal free energies (a.u.)	-1578.930988	-1578.931059	-1578.930668	-1578.930911

Table S9. DFT-optimized structures and thermodynamic parameters for low-energy conformers of **1D**

Conformers	Conf. A	Conf. B	Conf. C	Conf. D	Conf. E
DFT-optimized structures	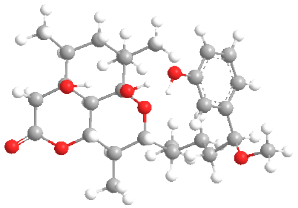	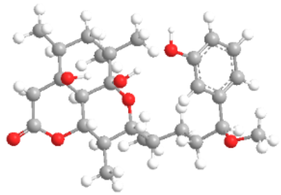	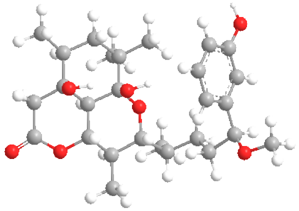	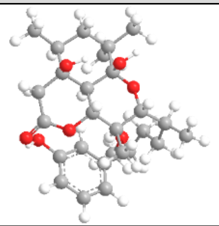	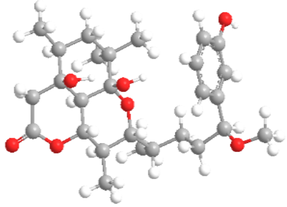
Population	12.30%	15.67%	10.15%	52.02%	9.86%
Total energy (a.u.)	-1579.53886739	-1579.53909570	-1579.53868676	-1579.54022736	-1579.53865869
Sum of electronic and zero-point energies (a.u.)	-1578.894092	-1578.893944	-1578.893869	-1578.894337	-1578.893825
Sum of electronic and thermal energies (a.u.)	-1578.860010	-1578.860183	-1578.859863	-1578.860931	-1578.859829
Sum of electronic and thermal enthalpies (a.u.)	-1578.859066	-1578.859239	-1578.858919	-1578.859987	-1578.858884
Sum of electronic and thermal free energies (a.u.)	-1578.958848	-1578.956756	-1578.957776	-1578.955622	-1578.957694

Table S10. DFT-optimized structures and thermodynamic parameters for low-energy conformers of **2A**

Conformers	Conf. A	Conf. B	Conf. C	Conf. D	Conf. E
DFT-optimized structures	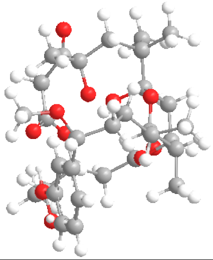	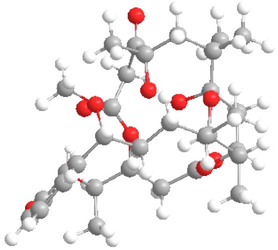	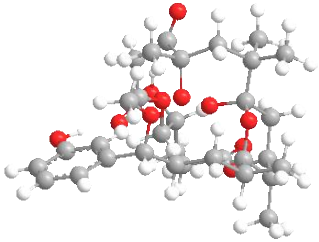	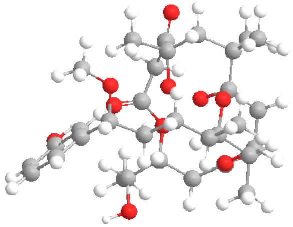	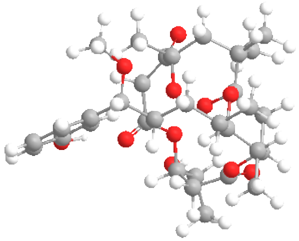
Population	3.06%	96.33%	0.28%	0.31%	0.02%
Total energy (a.u.)	-2074.50935574	-2074.51260892	-2074.50708429	-2074.50720925	-2074.50457360
Sum of electronic and zero-point energies (a.u.)	-2073.749474	-2073.753023	-2073.747338	-2073.748347	-2073.745112
Sum of electronic and thermal energies (a.u.)	-2073.707039	-2073.710597	-2073.704555	-2073.705107	-2073.702244
Sum of electronic and thermal enthalpies (a.u.)	-2073.706095	-2073.709653	-2073.703611	-2073.704162	-2073.701300
Sum of electronic and thermal free energies (a.u.)	-2073.821216	-2073.824875	-2073.819937	-2073.821240	-2073.817628

Table S11. DFT-optimized structures and thermodynamic parameters for low-energy conformers of **2B**

Conformers	Conf. A	Conf. B	Conf. C	Conf. D
DFT-optimized structures	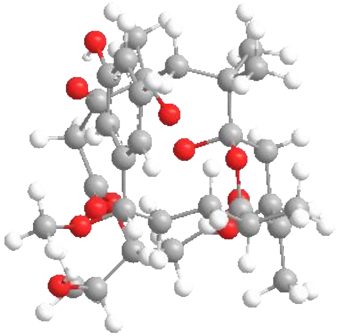	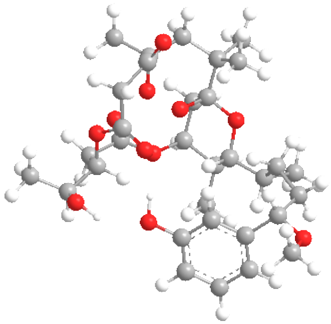	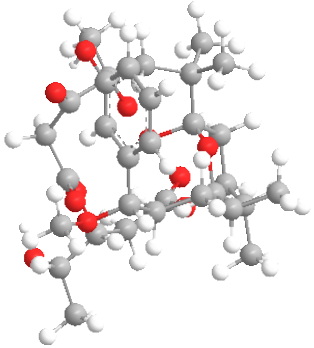	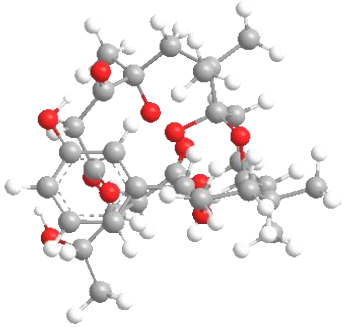
Population	8.01%	88.77%	2.45%	0.57%
Total energy (a.u.)	-2074.49868405	-2074.50095196	-2074.49756579	-2074.49618888
Sum of electronic and zero-point energies (a.u.)	-2073.739318	-2073.740694	-2073.738386	-2073.736944
Sum of electronic and thermal energies (a.u.)	-2073.696601	-2073.698403	-2073.695423	-2073.694119
Sum of electronic and thermal enthalpies (a.u.)	-2073.695657	-2073.697459	-2073.694479	-2073.693175
Sum of electronic and thermal free energies (a.u.)	-2073.812005	-2073.811809	-2073.811625	-2073.809634

Table S12. DFT-optimized structures and thermodynamic parameters for low-energy conformers of **2B**

Conformers	Conf. E	Conf. F	Conf. G
DFT-optimized structures	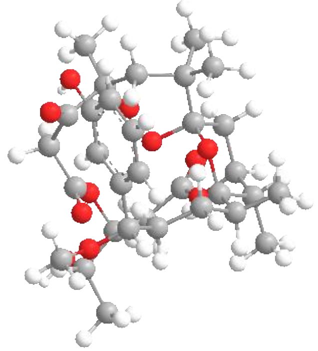	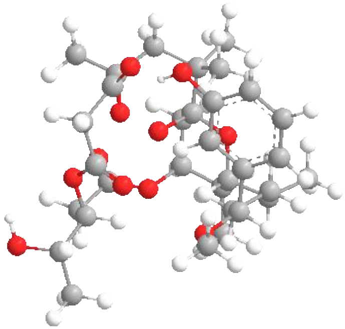	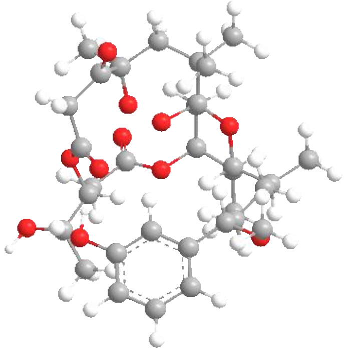
Population	0.11%	0.05%	0.04%
Total energy (a.u.)	-2074.49465127	-2074.49390286	-2074.49364628
Sum of electronic and zero-point energies (a.u.)	-2073.735903	-2073.735172	-2073.735605
Sum of electronic and thermal energies (a.u.)	-2073.692811	-2073.692065	-2073.692108
Sum of electronic and thermal enthalpies (a.u.)	-2073.691867	-2073.691120	-2073.691164
Sum of electronic and thermal free energies (a.u.)	-2073.809516	-2073.808811	-2073.810778

Table S13. DFT-optimized structures and thermodynamic parameters for low-energy conformers of **2C**

Conformers	Conf. A	Conf. B	Conf. C
DFT-optimized structures	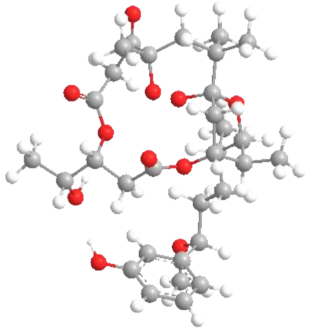	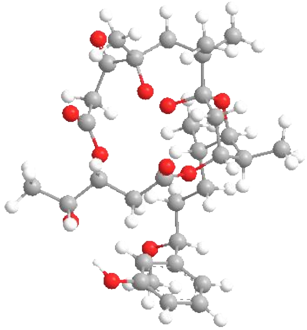	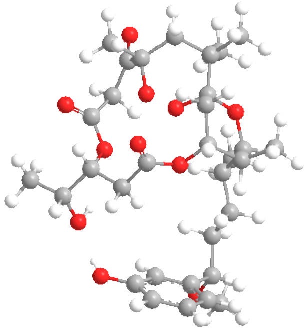
Population	65.08%	33.93%	0.99%
Total energy (a.u.)	-2074.49457781	-2074.49396364	-2074.49063066
Sum of electronic and zero-point energies (a.u.)	-2073.735215	-2073.734402	-2073.731494
Sum of electronic and thermal energies (a.u.)	-2073.692489	-2073.691737	-2073.688666
Sum of electronic and thermal enthalpies (a.u.)	-2073.691545	-2073.690793	-2073.687722
Sum of electronic and thermal free energies (a.u.)	-2073.807375	-2073.806820	-2073.803968

Table S14. DFT-optimized structures and thermodynamic parameters for low-energy conformers of **2D**

Conformers	Conf. A	Conf. B	Conf. C
DFT-optimized structures	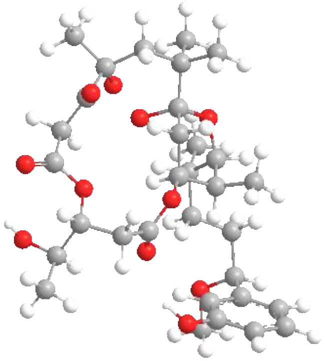	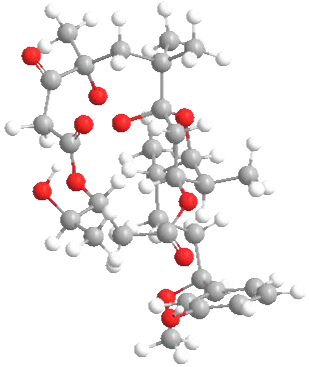	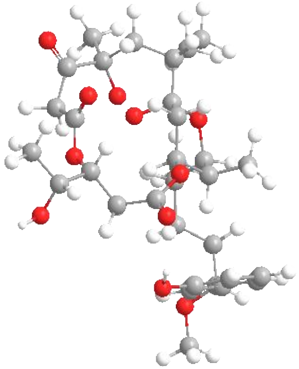
Population	78.60%	21.13%	0.27%
Total energy (a.u.)	-2074.47629938	-2074.47506068	-2074.47096012
Sum of electronic and zero-point energies (a.u.)	-2073.717043	-2073.715362	-2073.711361
Sum of electronic and thermal energies (a.u.)	-2073.673953	-2073.672690	-2073.668589
Sum of electronic and thermal enthalpies (a.u.)	-2073.673009	-2073.671746	-2073.667645
Sum of electronic and thermal free energies (a.u.)	-2073.791156	-2073.787842	-2073.784129

2. Figures

(A)

1		2	
C(μM)	Cell Viability (%)	C(μM)	Cell Viability (%)
control	100.00±4.79	control	100.00±4.05
1	86.70±0.97	1	94.80±2.66
5	83.93±6.25	2	93.22±3.64
10	79.69±5.38	4	56.70±2.22
20	51.71±4.05	6	31.46±1.27
30	40.11±1.61	8	22.89±1.81
40	33.98±1.10	10	18.42±1.54
50	11.34±0.86	20	12.74±1.01

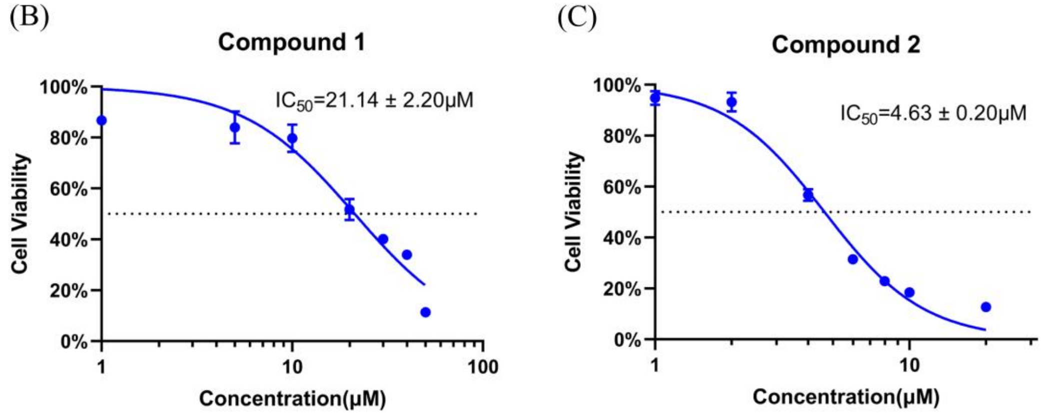

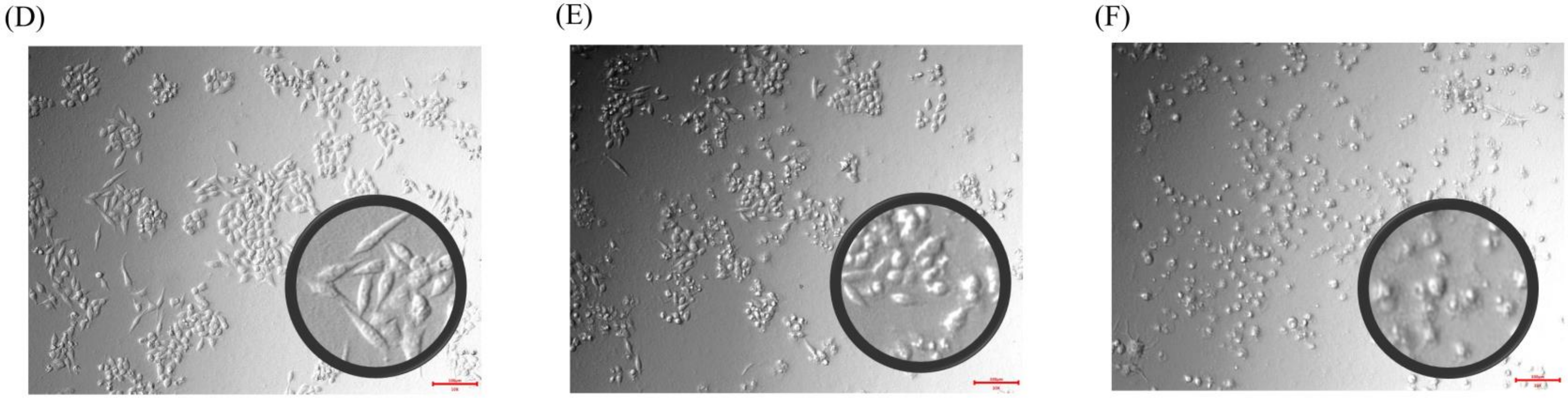

Figure S1. Dose-dependent effects of **1** and **2** on SW480 cell survival rate (72h).
(A) Dose-dependent effects of **1** and **2** on SW480 cell survival rate (72h). Values are demonstrated as mean ± SD from 4 independent experiments. (B) cell viability curve after teated with **1**. (C) cell viability curve after teated with **2**. (D) cell morphology of the control group. (E) cell morphology after teated with **1** (10 μM, 72h). (F) cell morphology after teated with **2** (10 μM, 72h).

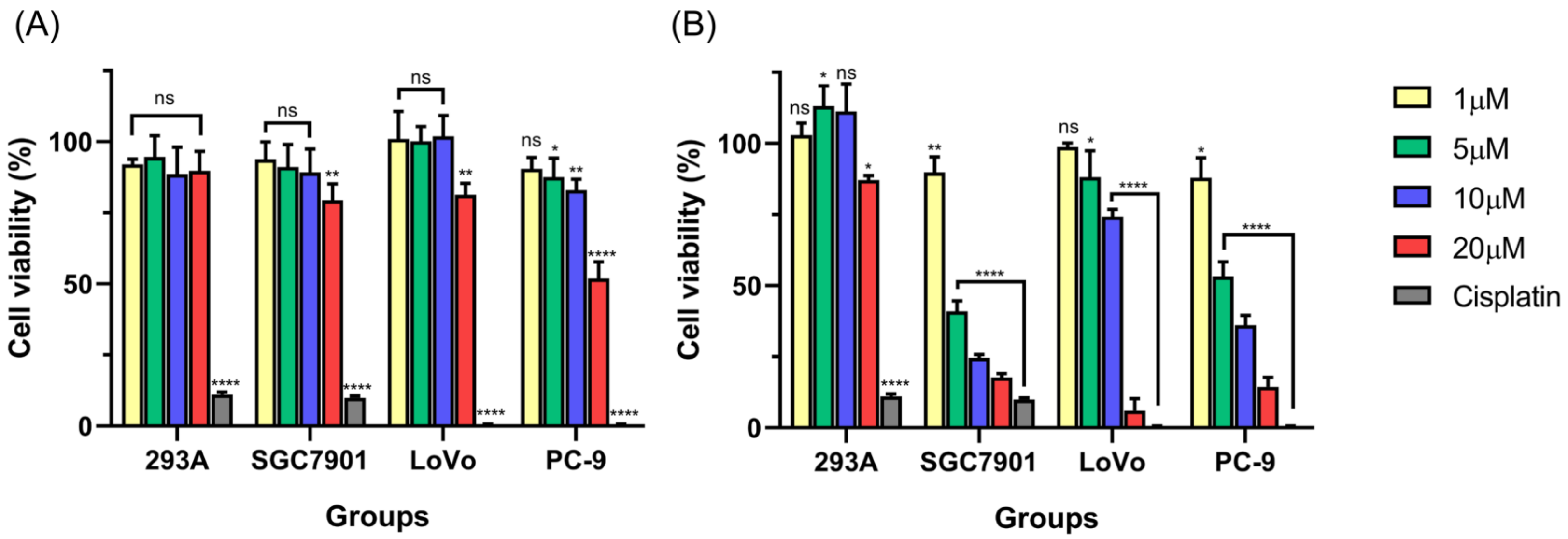

Figure S2. Cytotoxic effects of two compounds for 72 h on different cells as measured using a cell viability assay. Cisplatin (5 μM) was used as positive control. Asterisks indicate statistical significance at $p < 0.05$ and *ns* means no significant differences, compared to the untreated control group. The data were expressed as the means ± SD of four independent experiments. (A) Cytotoxicity against 4 different cell lines of **1**. (B) Cytotoxicity against 4 different cell lines of **2**.

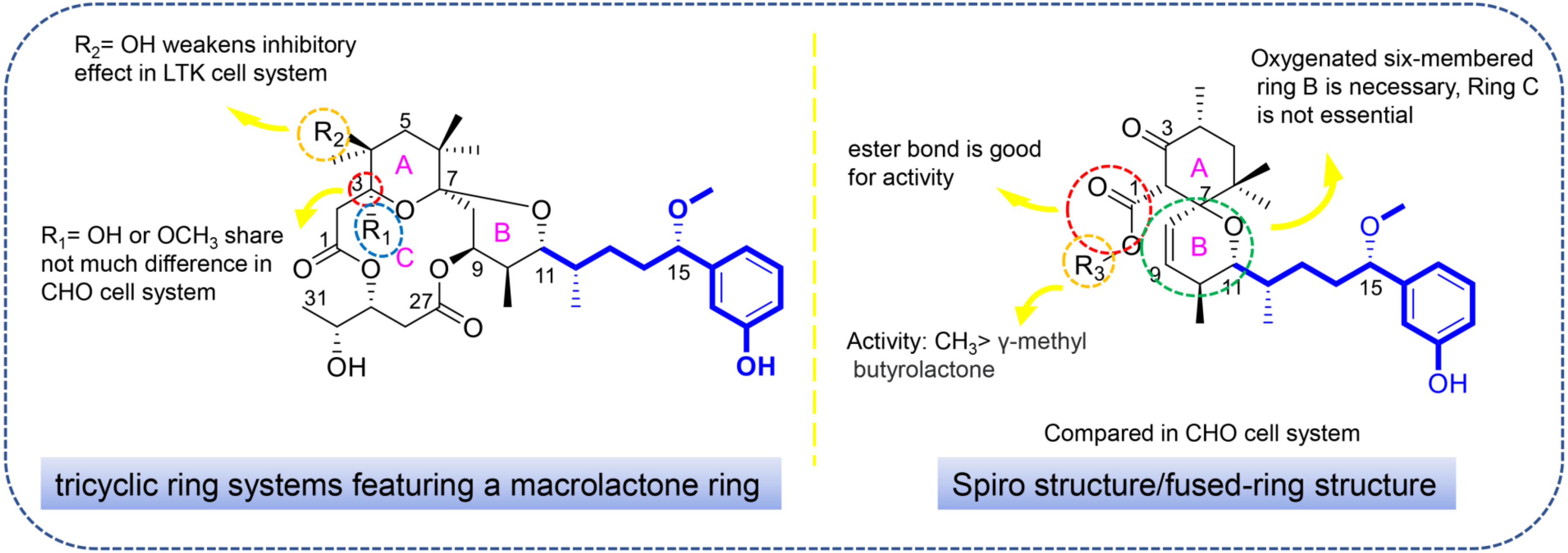

Figure S3. Critical structural features of ATXs leading to differential Kv1.5 inhibitory activities.

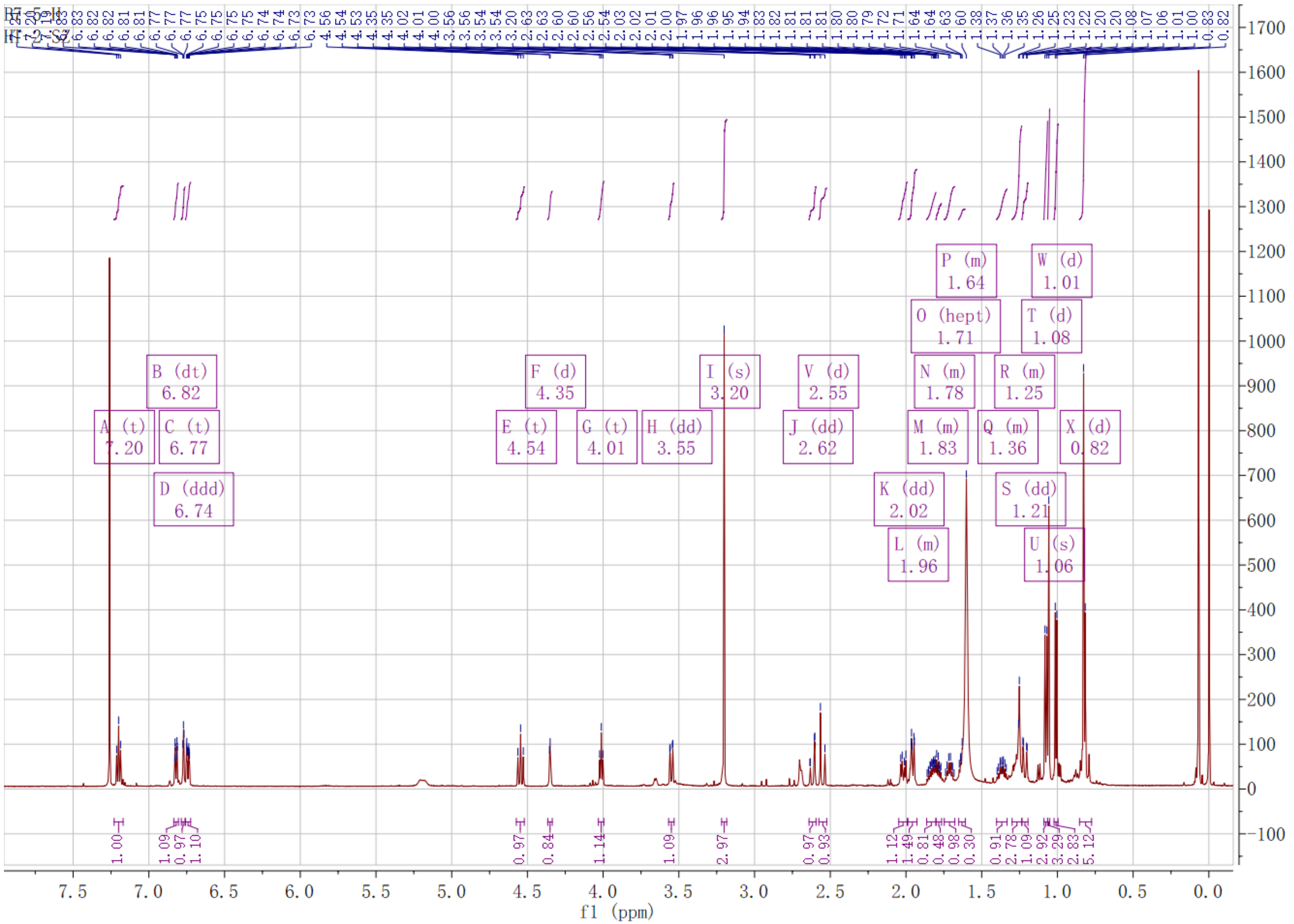

Figure S4. ^{1}H NMR spectrum of Compound **1** (600 MHz, $CDCl_3$)

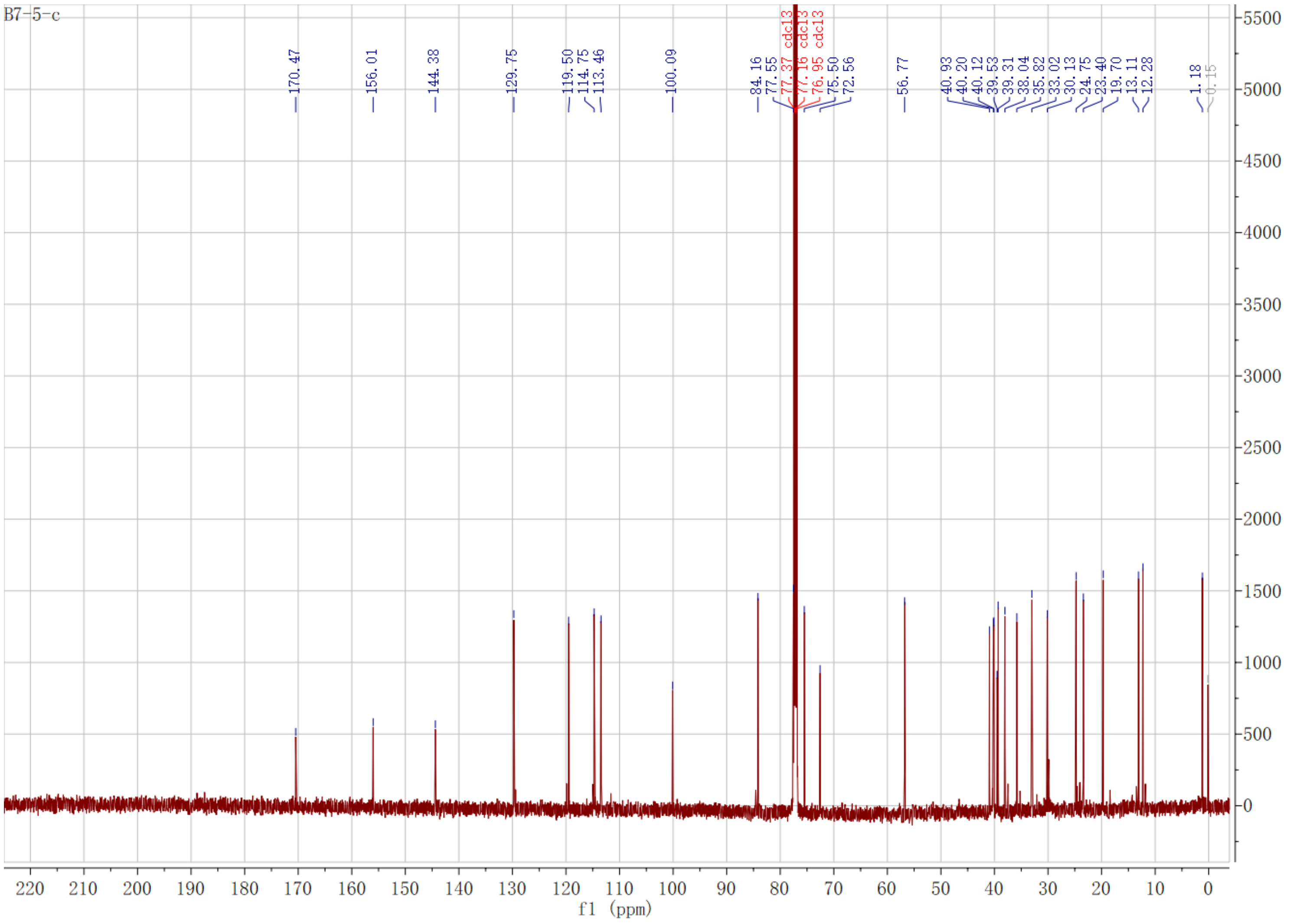

Figure S5. ^{13}C NMR spectrum of Compound **1** (150 MHz, $CDCl_3$)

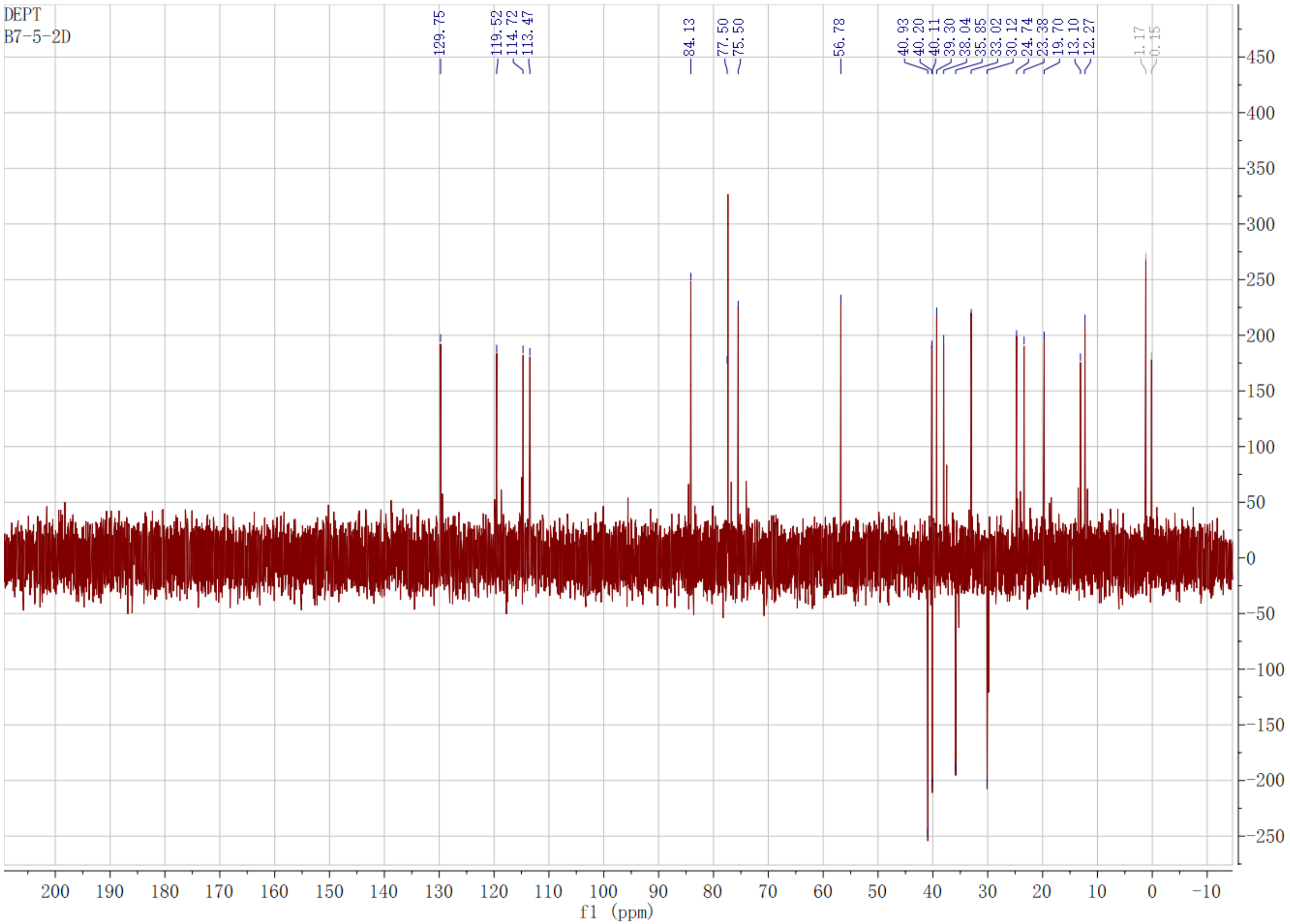

Figure S6. DEPT spectrum of Compound **1** (150 MHz, $CDCl_3$)

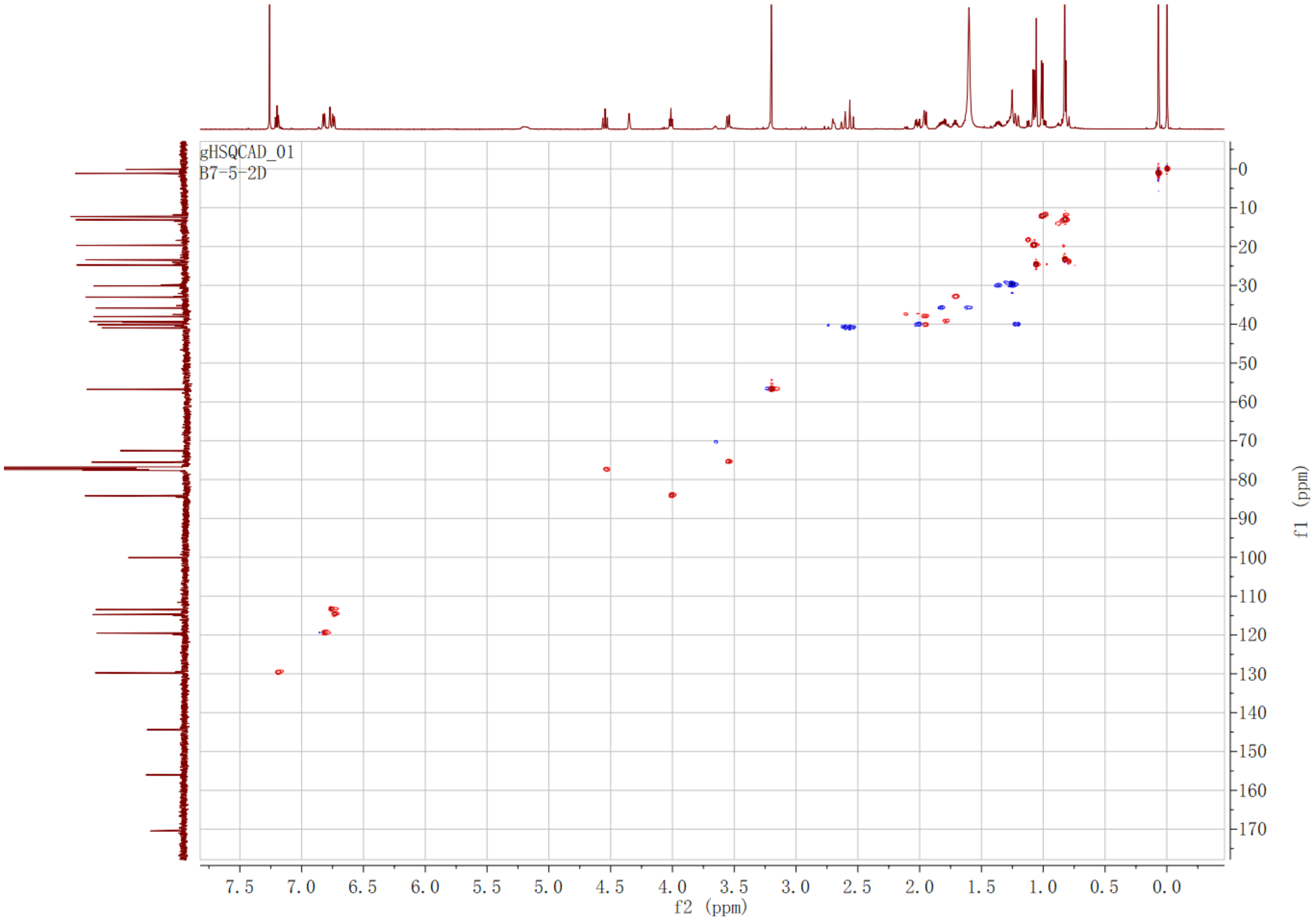

Figure S7. HSQC spectrum of Compound **1** (600 MHz, $CDCl_3$)

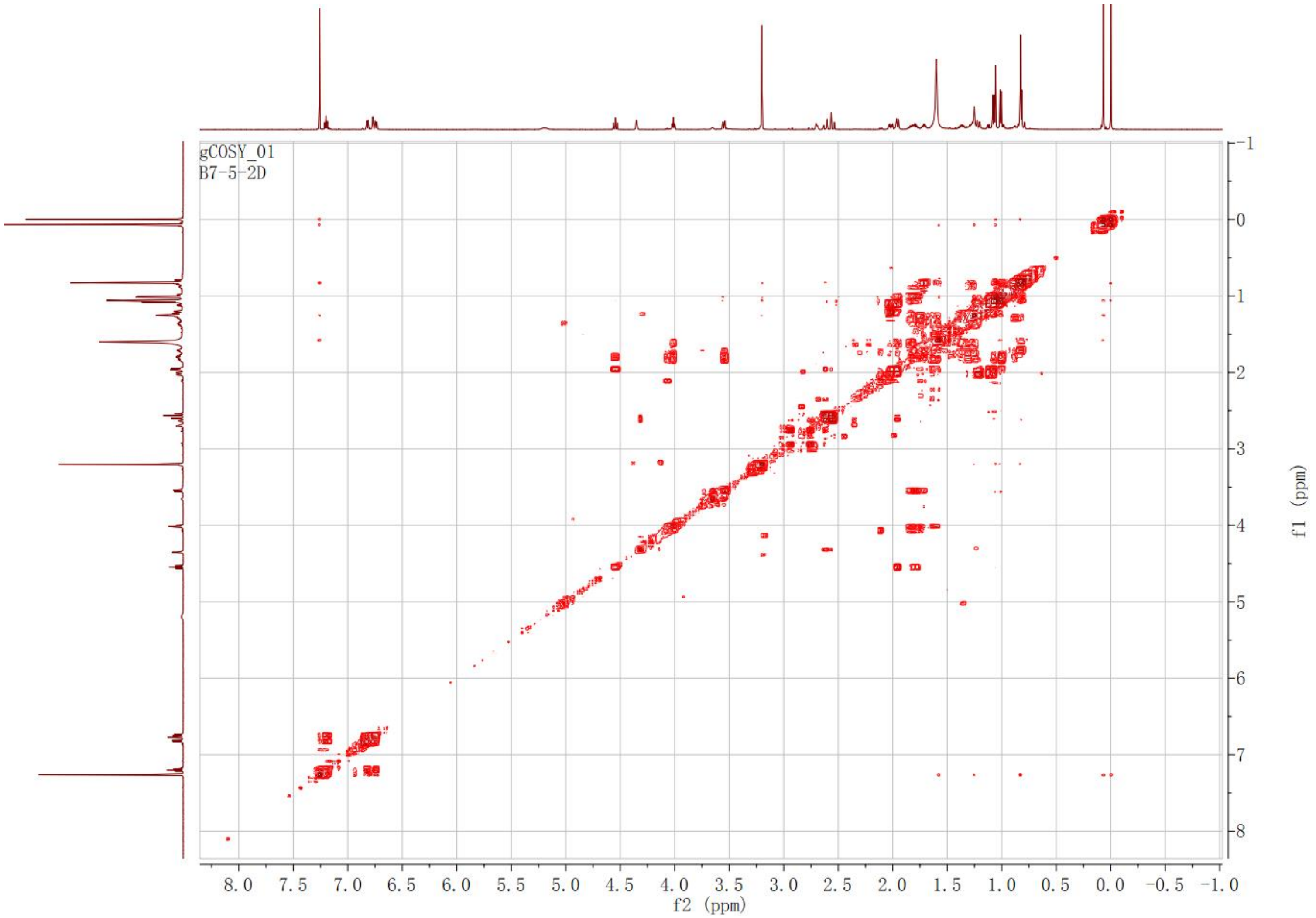

Figure S8. ^1H-^1H COSY spectrum of Compound **1** (600 MHz, $CDCl_3$)

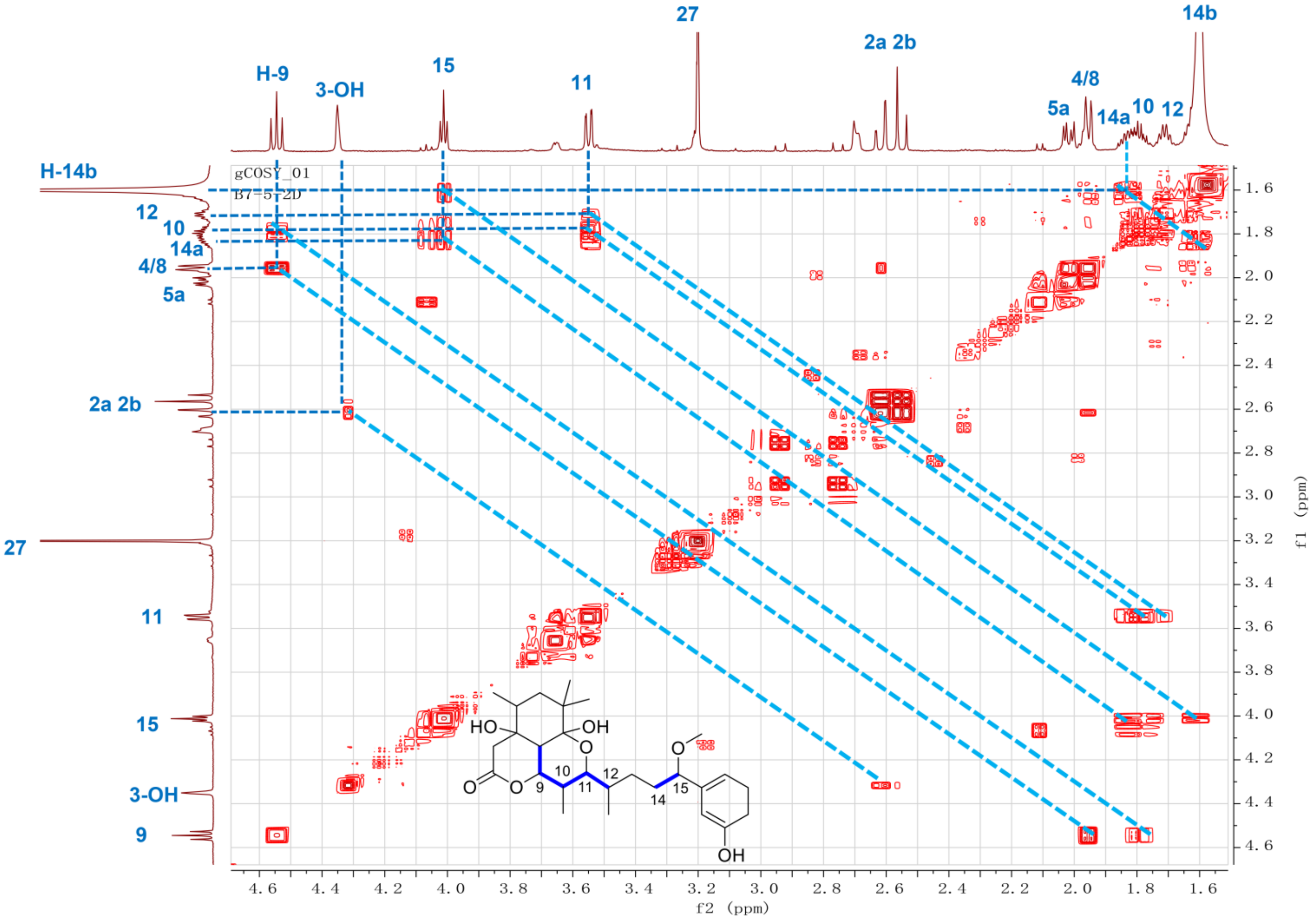

Figure S9. Expansion of ^{1}H-^{1}H COSY spectrum of Compound **1** (600 MHz, $CDCl_3$)

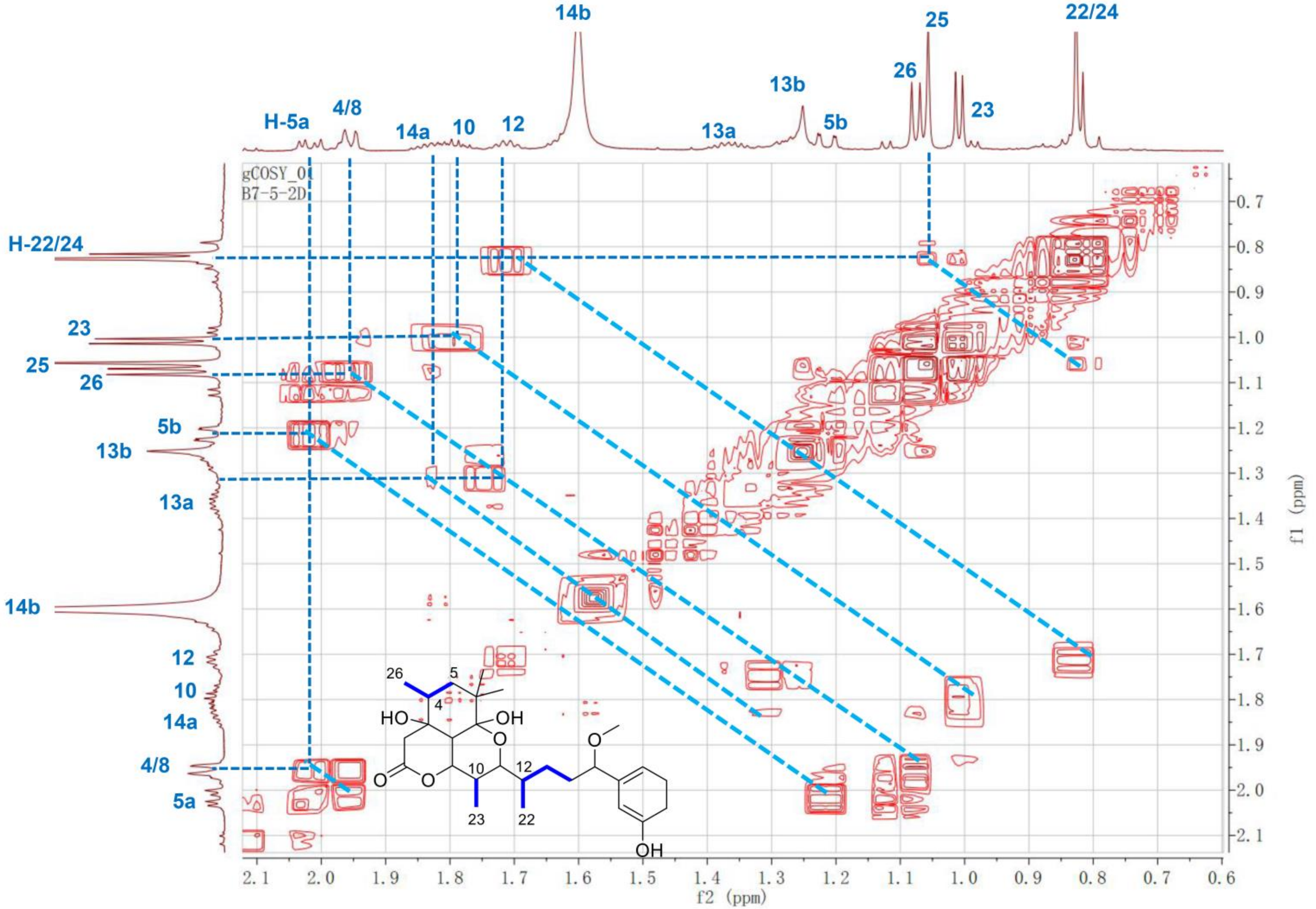

Figure S10. Expansion of ^{1}H-^{1}H COSY spectrum of Compound **1** (600 MHz, $CDCl_3$)

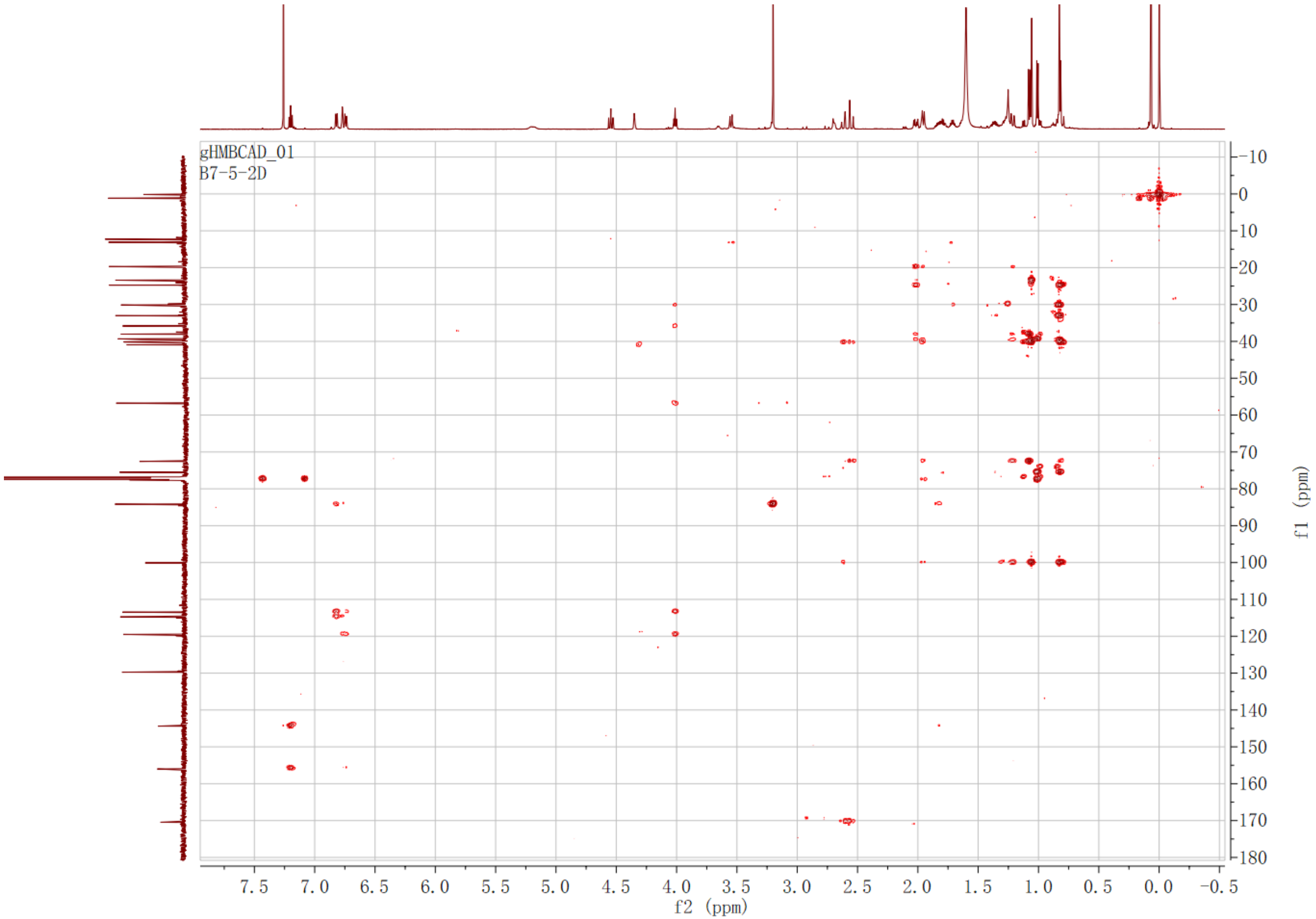

Figure S11. HMBC spectrum of Compound **1** (600 MHz, $CDCl_3$)

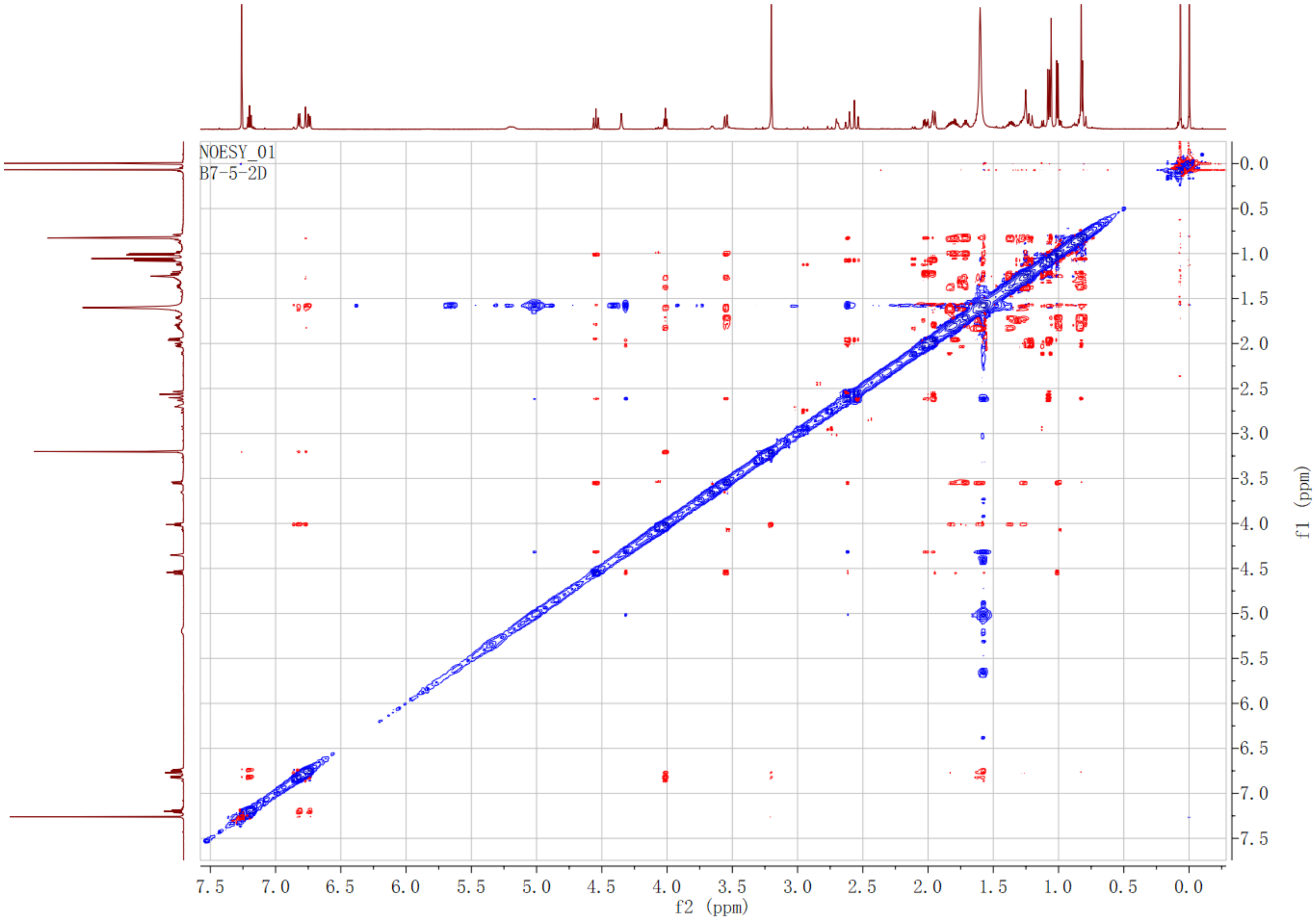

Figure S12. NOESY spectrum of Compound **1** (600 MHz, $CDCl_3$)

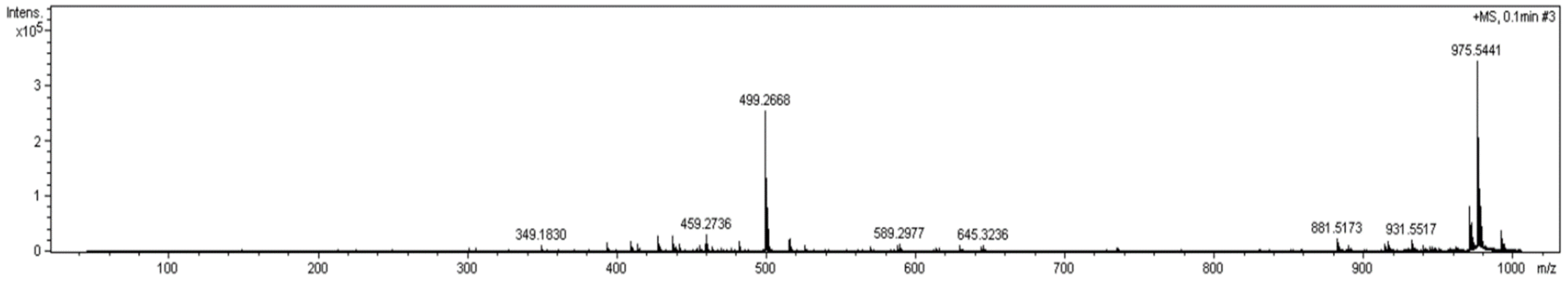

Figure S13. HRESIMS spectrum of Compound **1** in MeOH

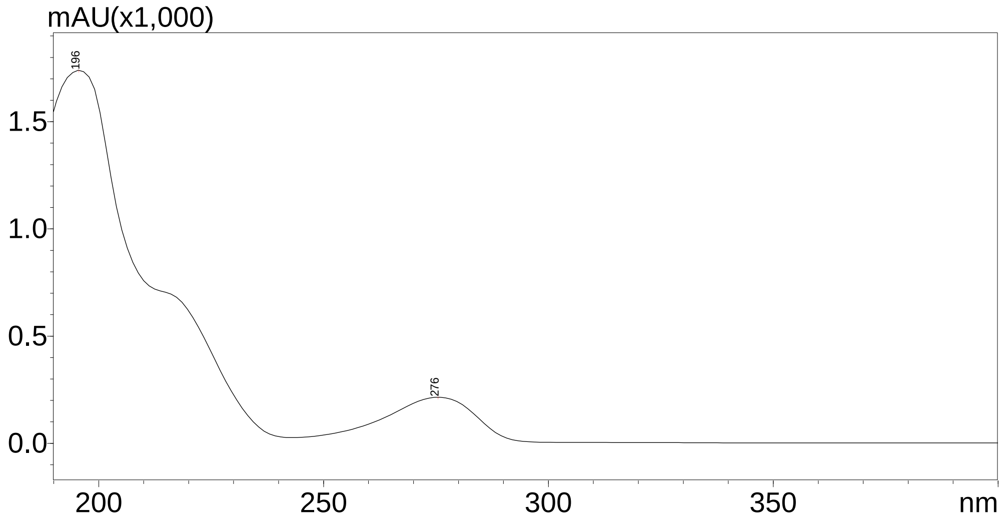

Figure S14. UV spectrum of Compound **1** in MeCN

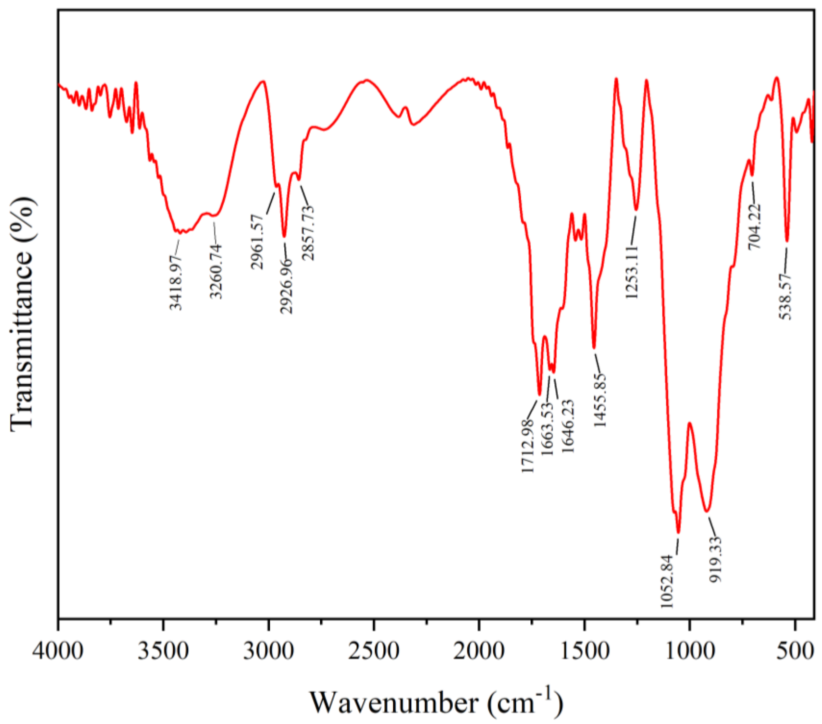

Figure S15. IR spectrum of Compound **1**

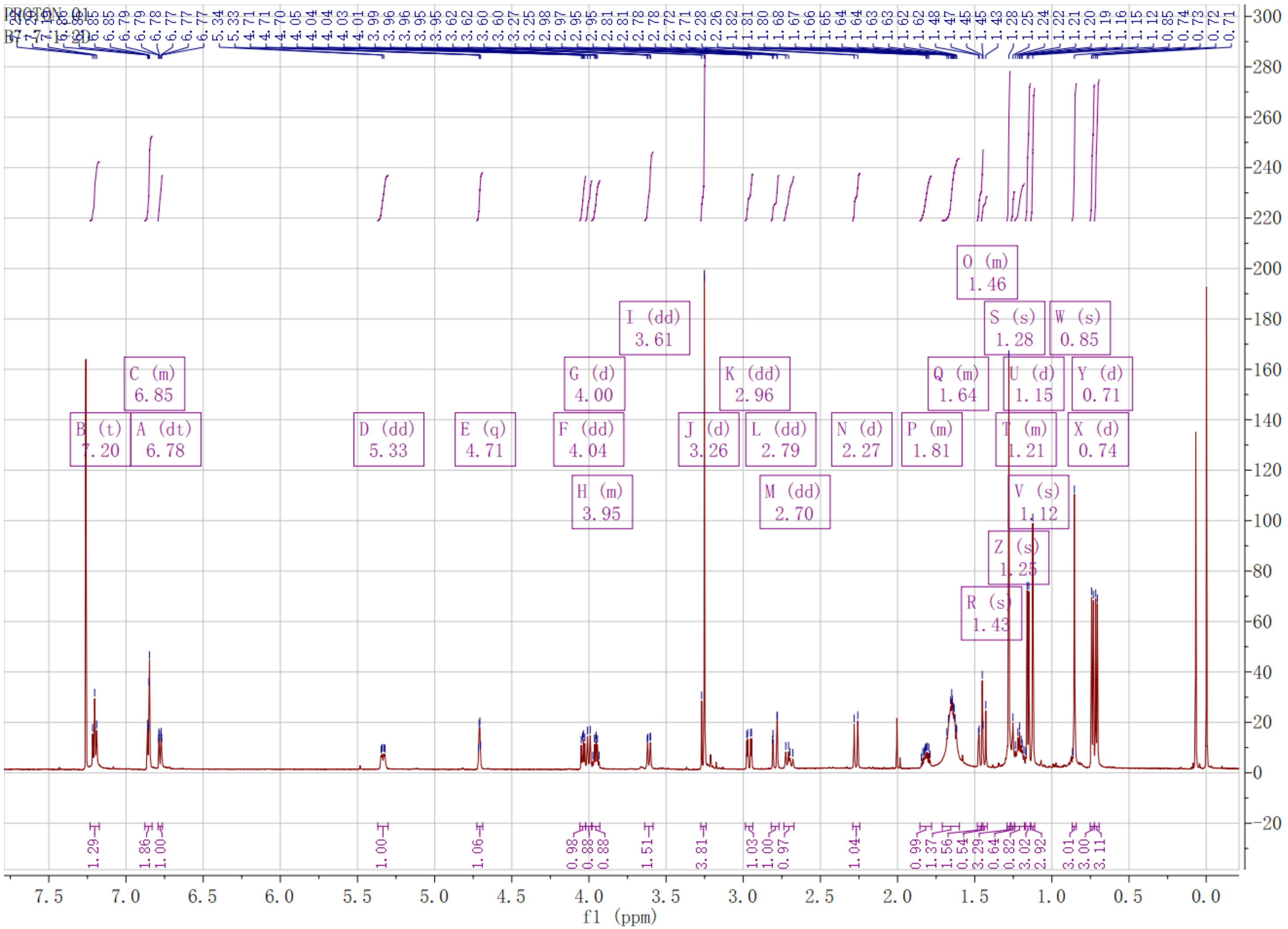

Figure S16. ^{1}H NMR spectrum of Compound **2** (600 MHz, $CDCl_3$)

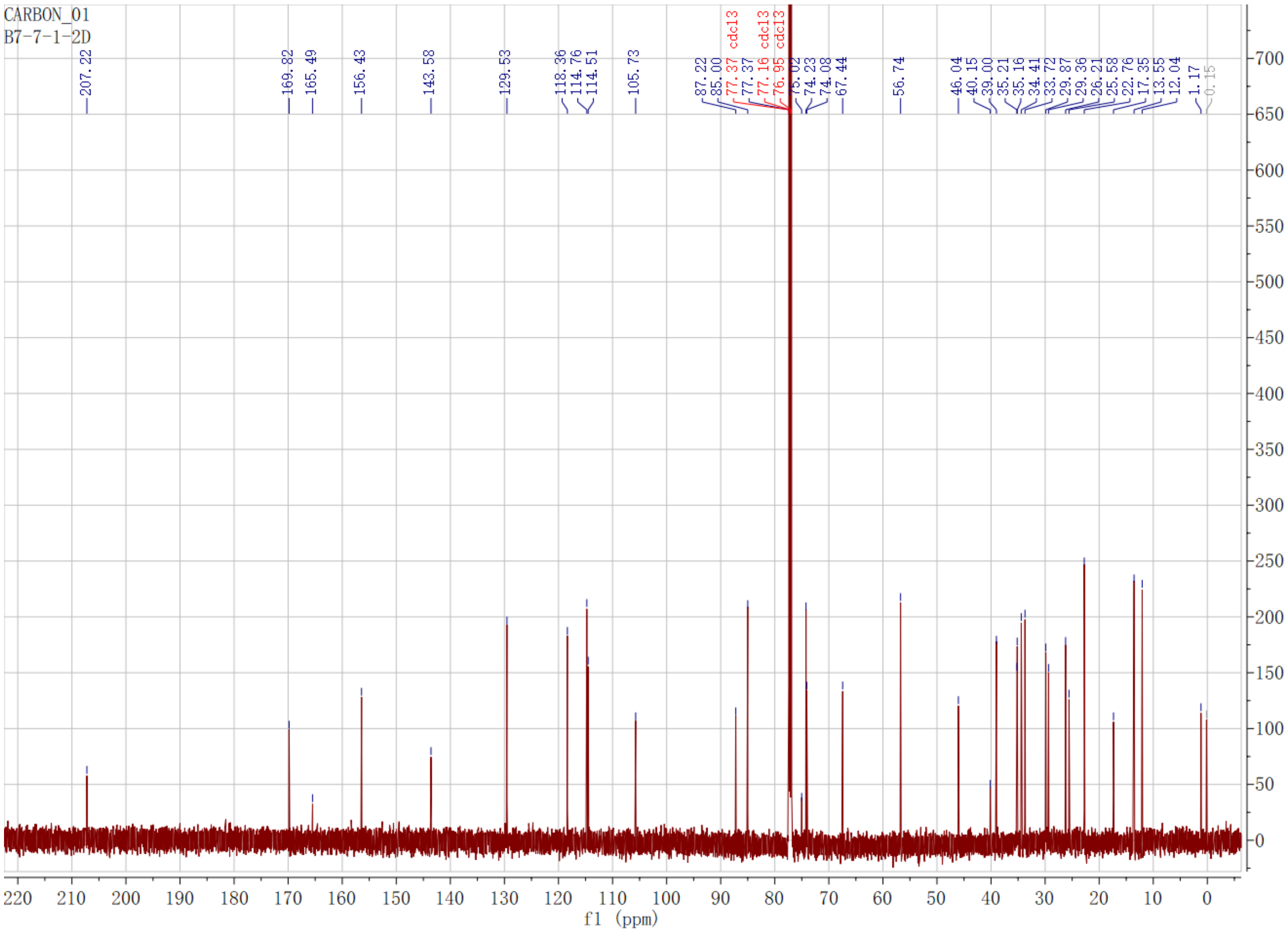

Figure S17. ^{13}C NMR spectrum of Compound **2** (150 MHz, $CDCl_3$)

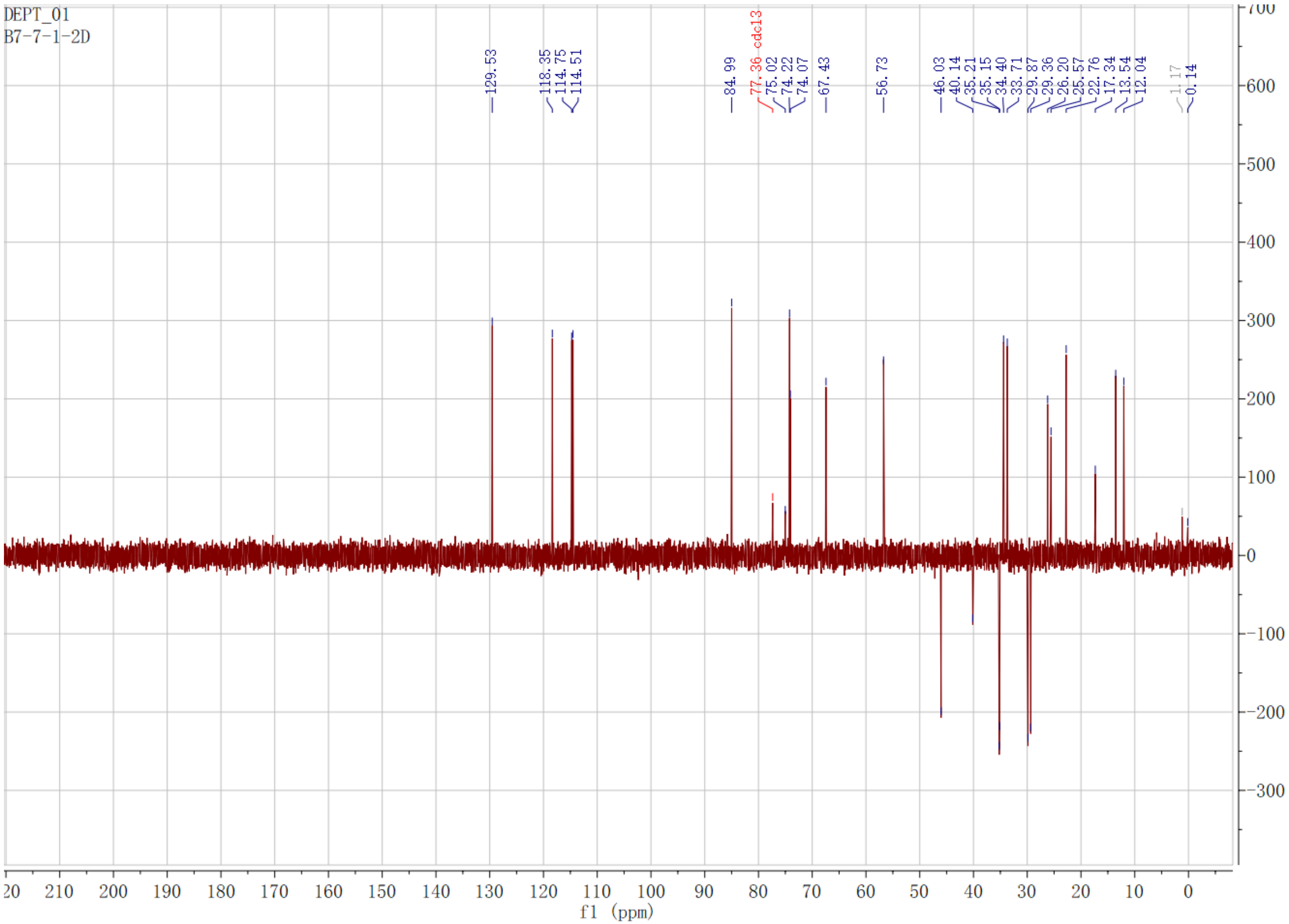

Figure S18. DEPT spectrum of Compound **2** (150 MHz, $CDCl_3$)

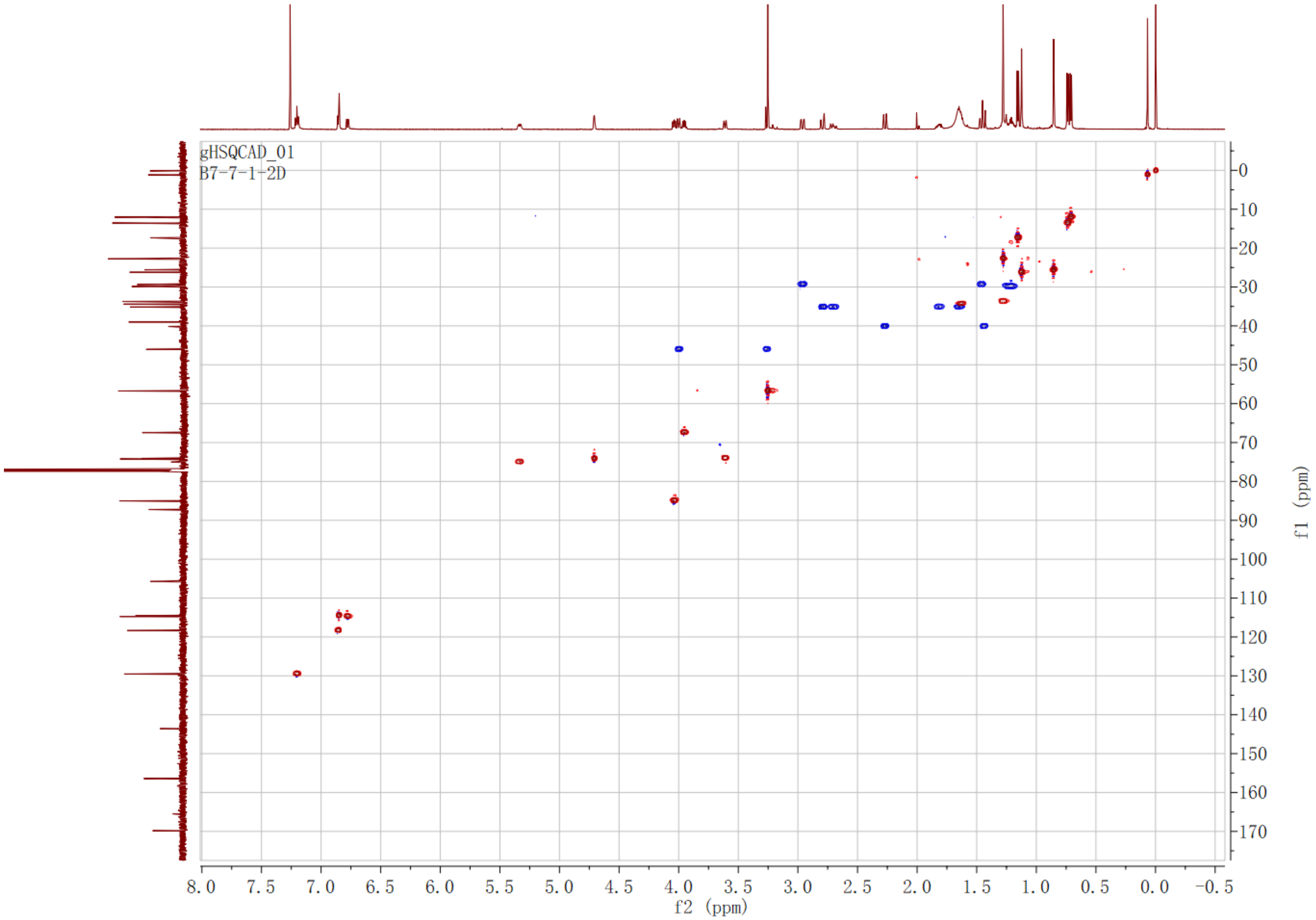

Figure S19. HSQC spectrum of Compound **2** (600 MHz, $CDCl_3$)

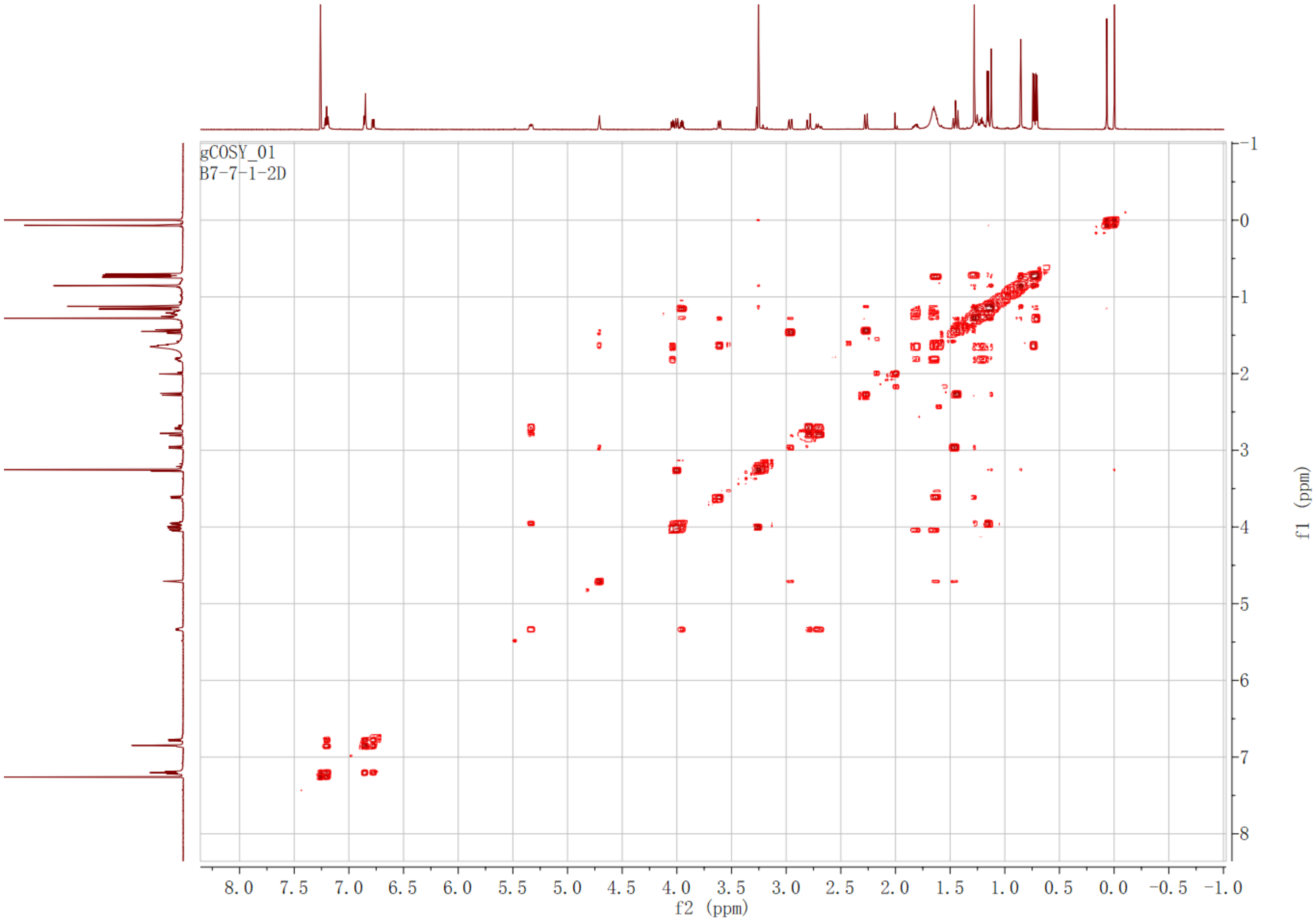

Figure S20. ^{1}H-^{1}H COSY spectrum of Compound **2** (600 MHz, $CDCl_3$)

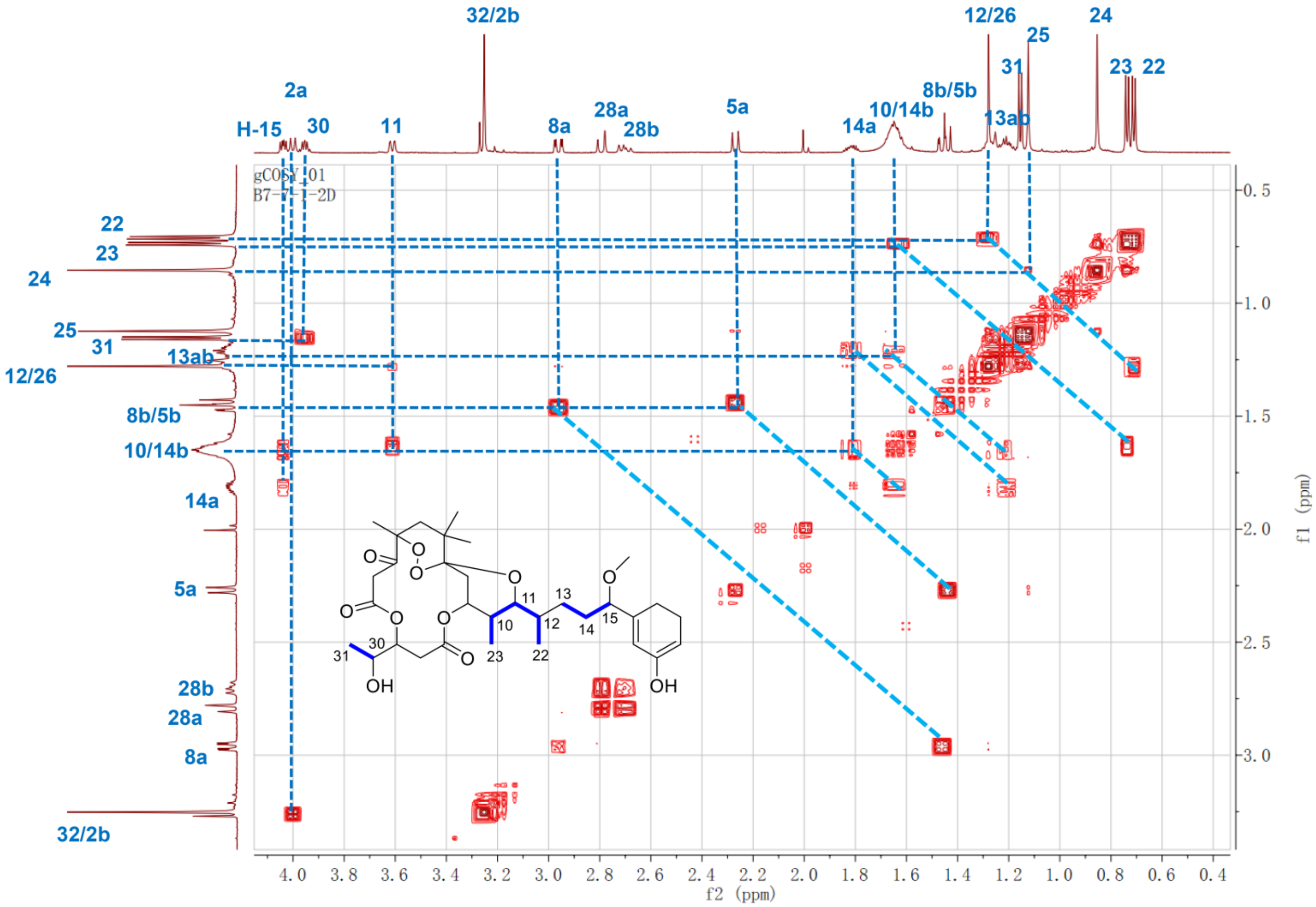

Figure S21. Expansion of ^{1}H-^{1}H COSY spectrum of Compound **2** (600 MHz, $CDCl_3$)

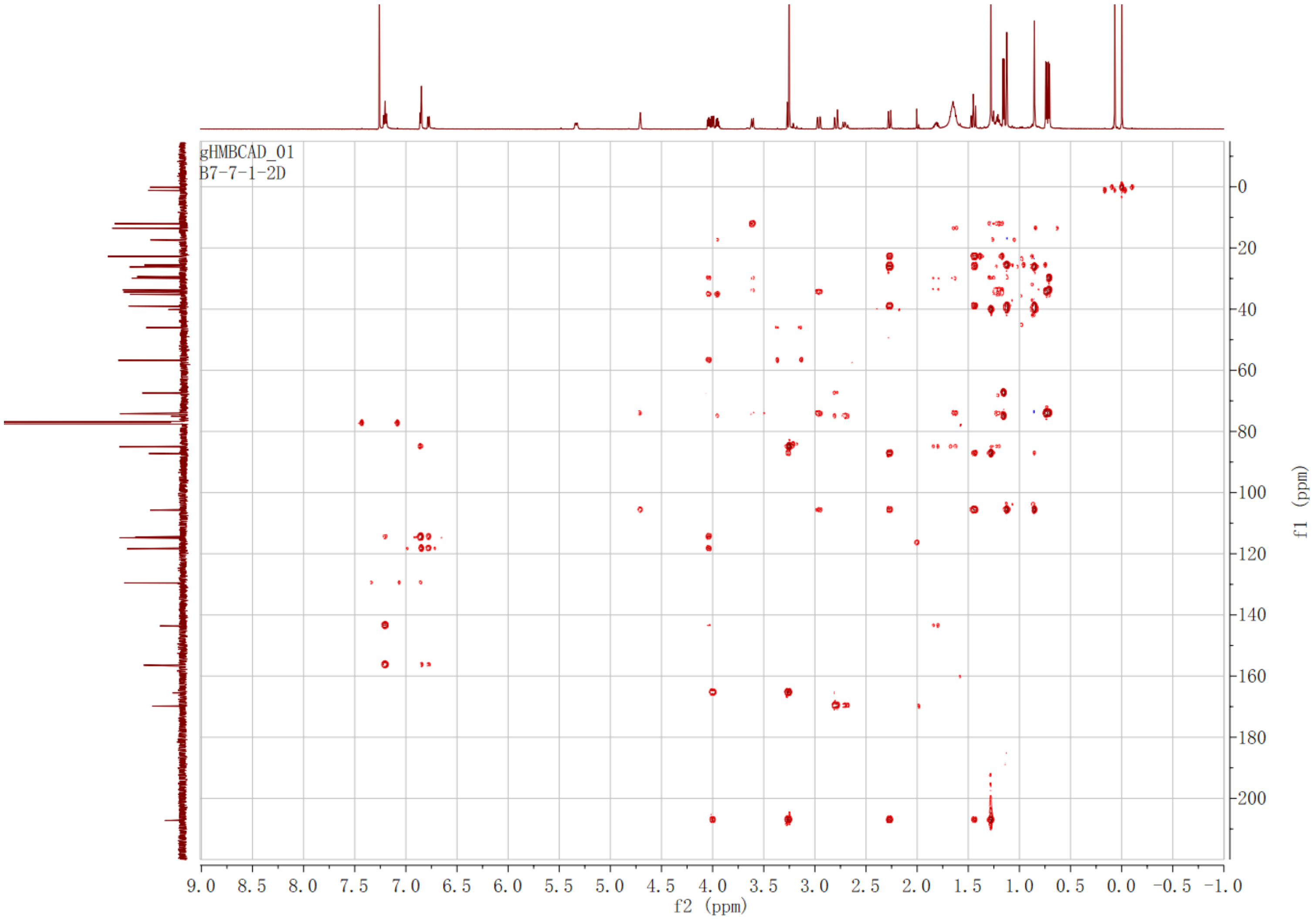

Figure S22. HMBC spectrum of Compound **2** (600 MHz, $CDCl_3$)

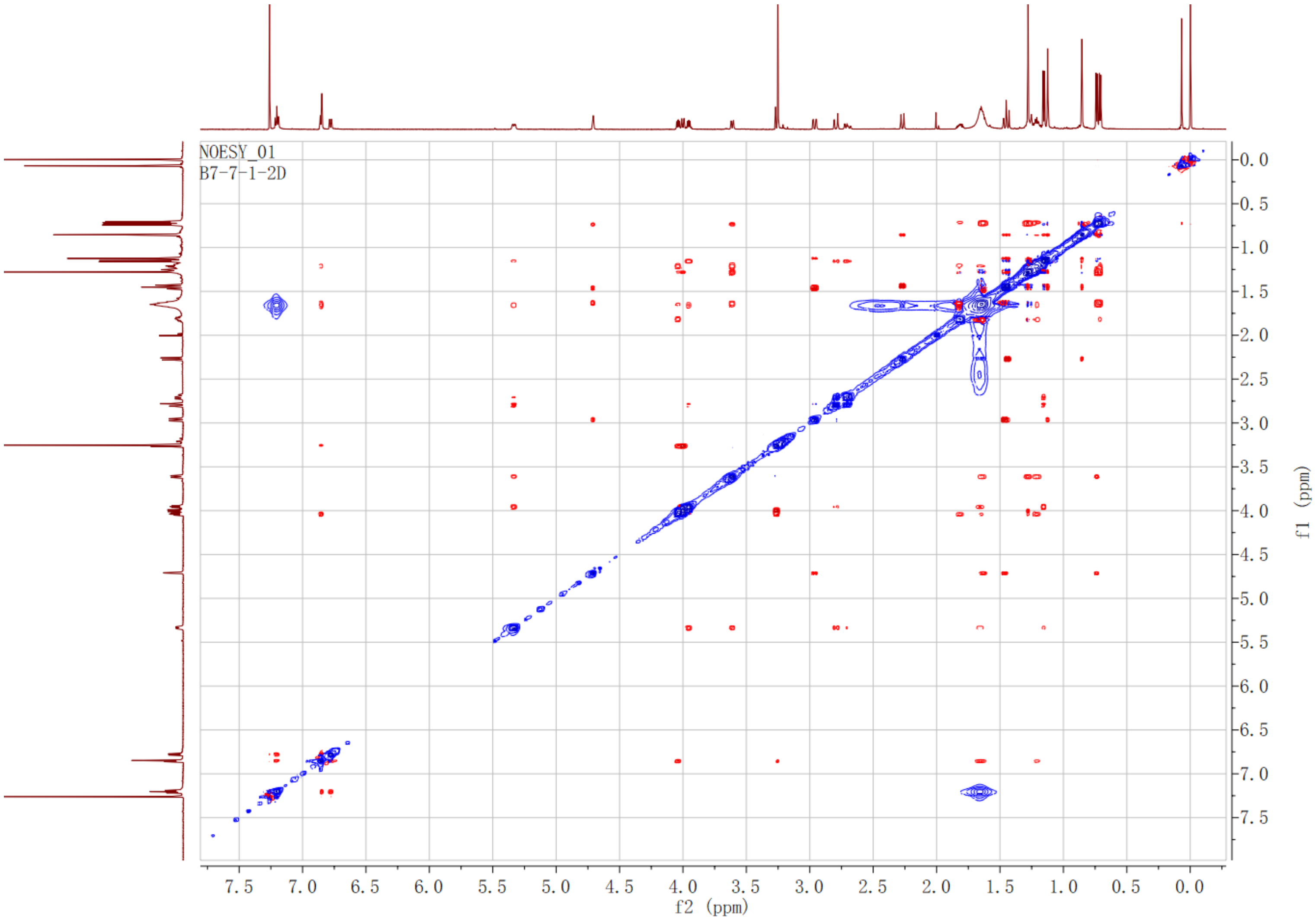

Figure S23. NOESY spectrum of Compound **2** (600 MHz, $CDCl_3$)

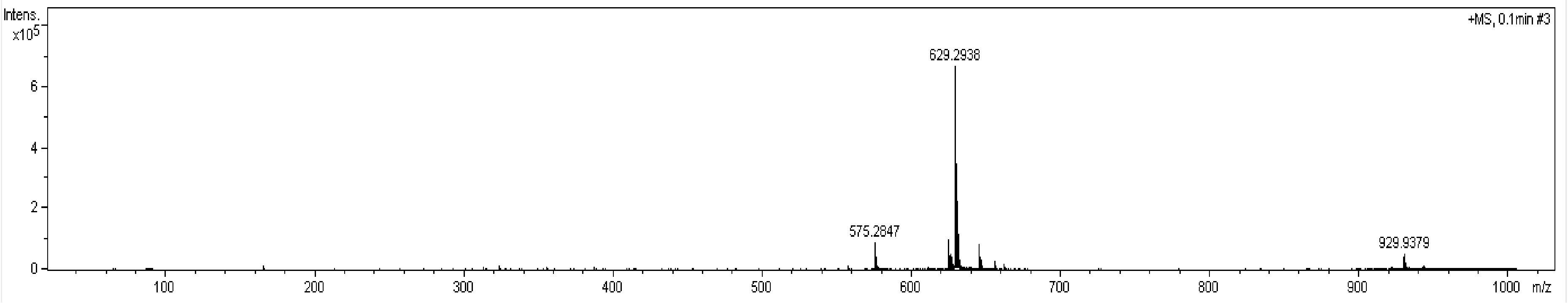

Figure S24. HRESIMS spectrum of Compound **2** in MeOH

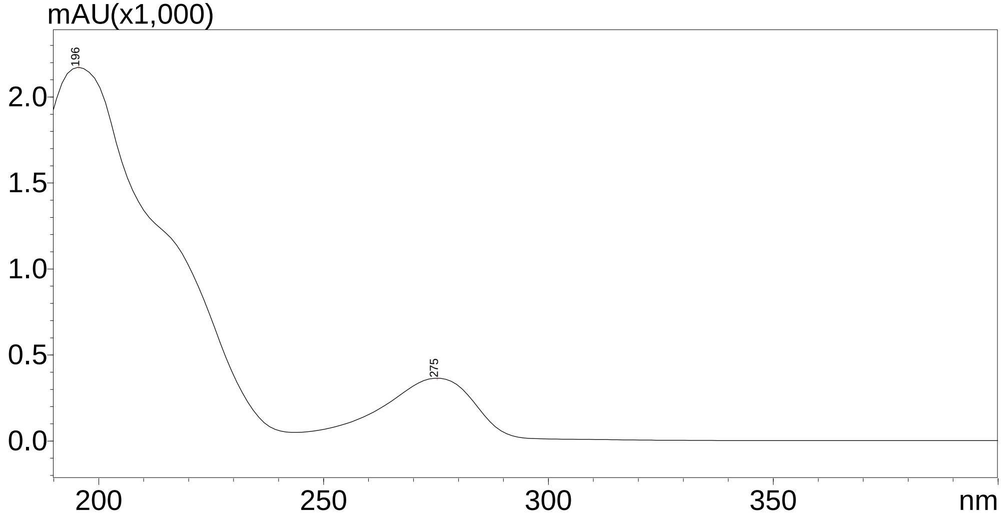

Figure S25. UV spectrum of Compound **2** in MeCN

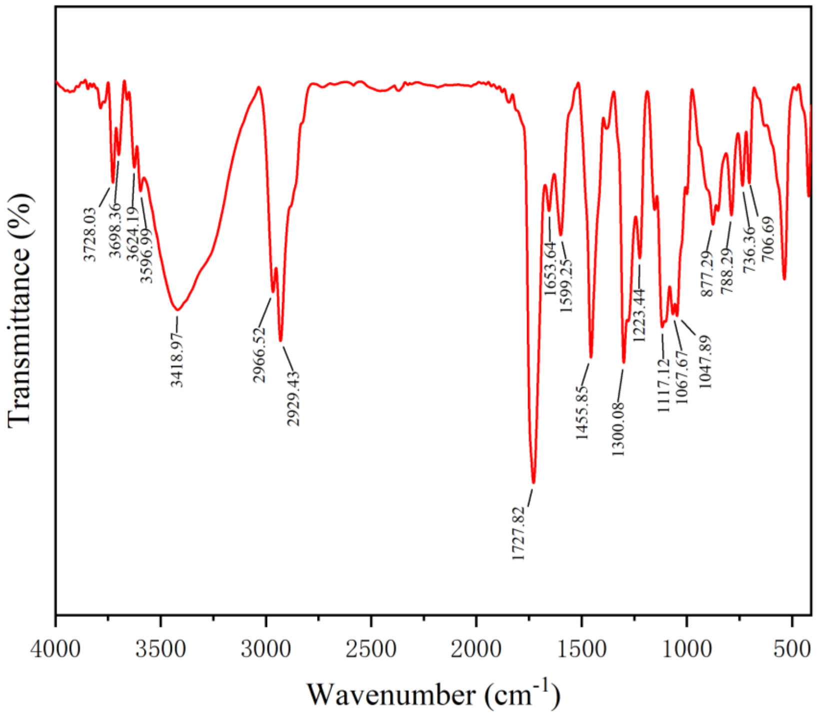

Figure S26. IR spectrum of Compound **2**

3. Morphological and molecular identification of cyanobacterium

The cyanobacteria strains used in this study were collected from Harbor of Hainan Sanya, China, Named as cyanobacterium HN. Colonies of cyanobacteria HN appeared as dark red, brown, or black tufts ranging from 15 to 25 cm in length and grew attached to sea rock and surface of the sea.

Filament width, cell width, and cell length of cyanobacteria HN were measured on the compound light microscope (Zeiss, Germany) with a 20x objective and 10x ocular lens with a calibrated optical micrometer. Filaments were long, of indeterminate length, 55-65 μm wide, formed by a uniseriate row of discoid cells encased in a firm, colorless, hyaline sheath which, when old, became yellowed and distinctly lamellated. Cells were discoid, 6-8 μm long, 30-40 μm broad, with rounded end cells without calyptra. Cell contents were finely granular without prominent granular inclusions (Figure S1.1(A)).

16S rDNA was used to characterize the identity of cyanobacterium samples. Total cyanobacterium genomic DNA from lyophilized samples was extracted by using TianGen Plant Genomic DNA Kit (TIANGEN Biotech Co., Ltd., Beijing, China) according to the manufacturer's instructions. Three PCR primer sets, CYA106F (5′-TACGGCTACCTTGTTAACGCGTGA-3′) / 781R (5′-GACTACTGGGGTATC- TAATCCCATT-3′), 27F (5′-AGAGTTTGATCCTGGCTCAG-3′) /809R (5′-GC-TTCGGCACGGCTCGGGTCGATA-3′) and MSR2F (5′-CGGTAATACGGGG- GAGGCAA-3′) /2R(5′-CCAACATCTCACGACACGAG-3′), were used for amplifying 16S rDNA. PCR reactions were performed in a BIO RAD Cycler C1000, according to the following profile: 5 min at 95 °C and 35 cycles of 30 s at 95°C, 1 min at 58 °C for CYA106F/781R, 30 s at 50 °C for27F/809R or MSR2F/2R, and 1 min at 72 °C, followed by 10 min at 72 °C. The products were analyzed by electrophoresis in 0.7% (w/v) agarose gels electrophoresis. 16S rDNA sequences of other cyanobacterial taxa were acquired from NCBI GenBank and EzBioCloud databases and aligned by using MUSCLE implemented in MEGA7.0, and the phylogenetic tree were reconstructed by MrBayes.

Cyanobacterium HN held the highest 16S rRNA gene similarity with *Lyngbya* sp. CENA128T with the value of 99%, revealing that cyanobacterium HN might belong to *Lyngbya* sp.. The phylogenetic trees based on the 16S rRNA gene sequences, reconstructed with the Bayesian MCMC methods, showed that cyanobacterium HN fell into the clade comprising *Lyngbya* species and formed a stable clade with *Lyngbya* sp. CENA128T (Figure S1.1(B)). According to these results, cyanobacteria HN belonged to *Lyngbya* sp.

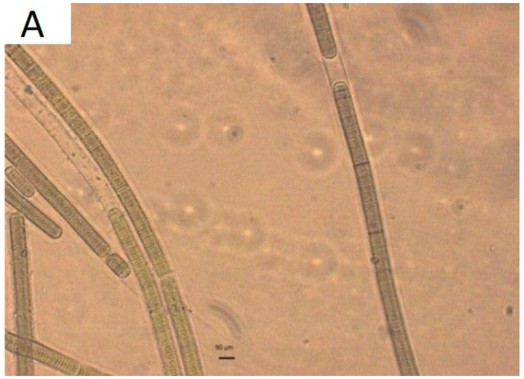

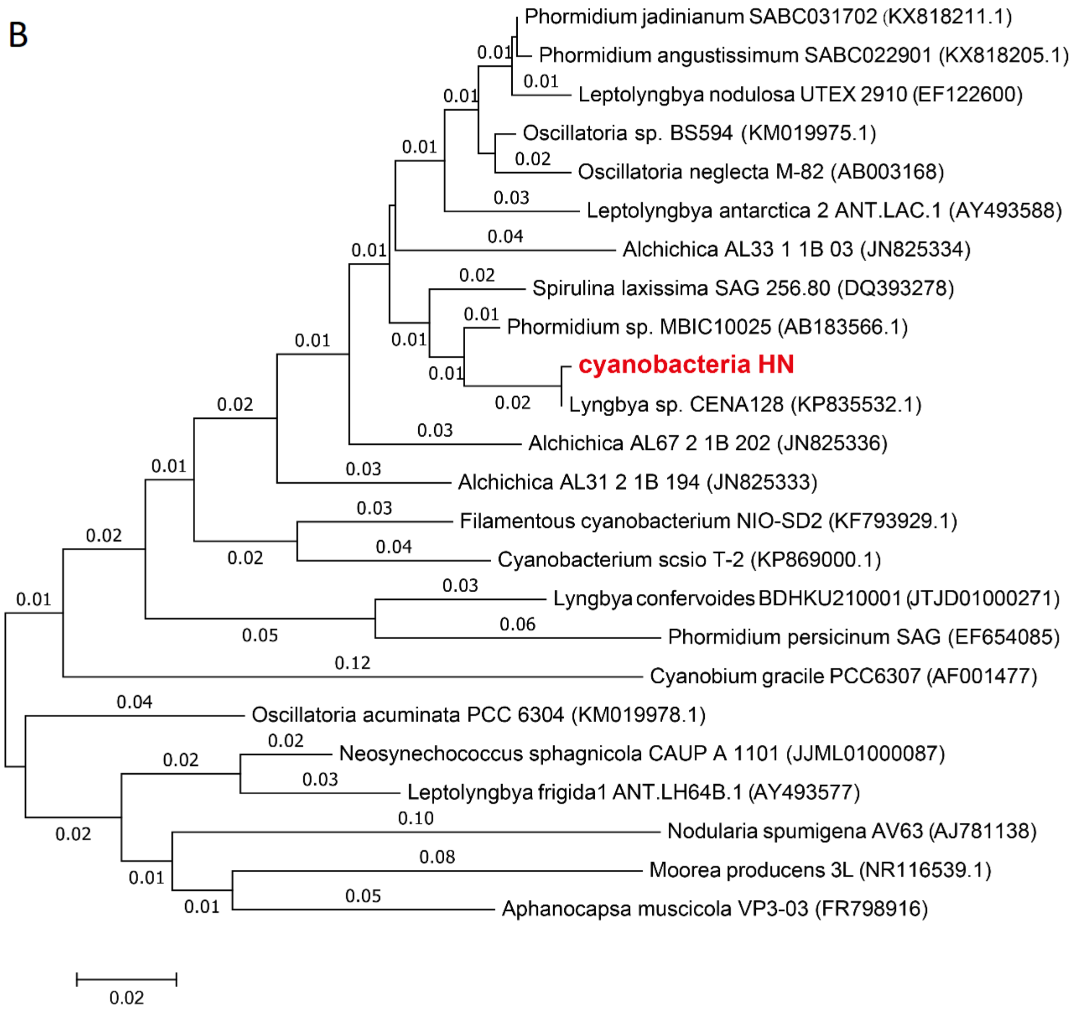

Figure S27. Morphological and molecular identification of cyanobacterium

(A) *Lyngbya* sp. collected in Harbor of Hainan. The specie, as identified based on morphological features, is shown in light micrographs. (B) Bayesian phylogenetic tree of *Lyngbya* sp. HN and its close relatives. Bootstrap was set as 20,000 replicates. Bar, 0.02 substitutions per nucleotide position.
